# Sex‐Specific Cardiovascular Consequences of Long‐Term High‐Salt Diet in Mice

**DOI:** 10.1161/JAHA.125.041945

**Published:** 2025-10-10

**Authors:** João M.N. Duarte, Sevilay Sahoglu‐Göktas, Lotte Vanherle, Zeinab Rafiee, Sneha Prakash, Kerstin Stemmer, Karl Swärd, Martin Magnusson, Anja Meissner

**Affiliations:** ^1^ Department of Experimental Medical Science, Faculty of Medicine Lund University Lund Sweden; ^2^ Wallenberg Center for Molecular Medicine Lund University Lund Sweden; ^3^ Division of Physiology & Vascular Biology, Institute of Theoretical Medicine, Faculty of Medicine University of Augsburg Germany; ^4^ Division of Molecular Cell Biology, Institute of Theoretical Medicine, Faculty of Medicine University of Augsburg Germany; ^5^ Department of Clinical Sciences Lund University Malmö Sweden; ^6^ Department of Cardiology Skåne University Hospital Malmö Sweden; ^7^ Hypertension in Africa Research Team (HART) North‐West University Potchefstroom South Africa

**Keywords:** cardiac remodeling, dietary intervention, salt‐sensitive hypertension, sex differences, vascular function, Animal Models of Human Disease, Basic Science Research, Physiology, Hypertension, Remodeling

## Abstract

**Background:**

Excessive dietary salt intake is associated with elevated blood pressure and damage to organs including the heart, vasculature, and kidneys. Women are more prone to salt‐sensitive hypertension, yet preclinical studies often focus on men.

**Methods:**

To examine sex‐specific responses to chronic salt loading, male and female C57Bl6/J mice were fed a high‐salt diet (HSD; 8% NaCl) from 7 to 14 months of age. Subsets of HSD mice were switched to a normal diet after 28 weeks. Blood pressure was measured via tail‐cuff plethysmography, and cardiac function assessed by magnetic resonance imaging. Vascular responses were analyzed by wire myography, and histological and molecular analyses were performed on heart, aorta, and kidney tissues.

**Results:**

After 56 weeks, only HSD‐fed females exhibited increased systolic blood pressure (*P*=0.015), whereas HSD‐fed males showed elevated left ventricular stroke volume, end‐diastolic volume, and mass (*P*<0.05). Males displayed aortic remodeling with increased wall thickness and synthetic smooth muscle marker expression. Mesenteric arteries had impaired contractile responses in males, whereas α1‐adrenergic tone was elevated in HSD‐fed females. Despite no overt renal injury, renal vascular thickening was observed in HSD males and glomerulosclerosis in normal diet females. Diet reversal normalized blood pressure in females and reversed cardiac changes in males.

**Conclusions:**

Chronic high salt intake leads to distinct sex‐specific cardiovascular remodeling in mice. Importantly, diet reversal mitigates these effects, highlighting the potential of dietary interventions in salt‐sensitive cardiovascular risk.

**Nonstandard Abbreviations and Acronyms**
HSD: high‐salt diet
KCl: potassium chloride
LVM: left ventricular mass
ND: normal diet
OPN: osteopontin
REV: diet reversal
SMA: smooth muscle actin

**Research Perspective**

**What Is New?**

- This study reveals sex‐specific cardiovascular responses to prolonged high‐salt intake in C57BL/6J mice, with females developing salt‐sensitive hypertension and males exhibiting cardiac and vascular remodeling independent of blood pressure changes, suggesting distinct physiological adaptations to chronic salt exposure between sexes.

**What Question Should be Addressed Next?**

- Understanding sex‐specific mechanisms of salt‐sensitive cardiovascular disease could improve risk stratification and lead to more targeted treatment strategies beyond blood pressure control, particularly in populations with high salt sensitivity.
- Future research should explore the molecular and hormonal drivers of these sex‐specific responses, particularly the roles of renal sodium handling, autonomic regulation, and aldosterone signaling in salt‐induced cardiovascular remodeling.

According to the World Health Organization, most people consume too much salt—on average 9 to 12 g/day, which is around twice the recommended maximum level of intake.^1^ Considerable evidence links excess dietary salt intake with the development of hypertension, left ventricular (LV) hypertrophy, and increased risk of stroke and coronary heart disease.^2^, ^3^ Recent preclinical and clinical data support that even in the absence of a blood pressure increase, excess dietary salt can adversely affect target organs, including the blood vessels, heart, kidneys, and brain.^4^

Among patients with essential hypertension, dietary salt consumption is a causative factor in 50% to 80% of cases.^5^ The overarching present clinical notion is that there are subsets of patients who are “salt sensitive” whose blood pressure increases or decreases with high or low salt consumption, respectively.^6^ The concept of salt‐sensitive hypertension is not a new phenomenon. However, emerging data on the mechanisms begin to shed light on the clinical presentation of salt sensitivity, notably its regulation by biological sex. Large population studies indicate that salt‐sensitive changes in blood pressure are increased in women both in amplitude of salt‐induced changes in blood pressure and in overall prevalence of a change in blood pressure in response to dietary salt.^7^ Despite this evidence, numerous clinical and experimental studies demonstrate that renal salt‐mediated hypertension mechanisms predominate in men and male animals. Male‐specific mechanisms of salt‐sensitive hypertension appear to involve dysfunctional renal physiology.^8^ On the contrary, emerging evidence indicates that aldosterone production is sex‐specifically heightened in salt‐sensitive hypertensive women and female rodent models, which may be regulated by intra‐adrenal renin‐angiotensin system activation^9^ and sex hormone receptors.^10^, ^11^ However, most studies investigating the mechanisms underlying salt‐induced increases in blood pressure are conducted on young male rodents. To the best of our knowledge, there is hitherto no study demonstrating how excessive salt intake affects the entire cardiovascular system (including the heart and different vascular beds) over the span of adult life and how sex‐specific responses evolve.

To address this gap, the current study systematically examines the long‐term effects of high salt exposure on the cardiorenal system of male and female C57BL/6J mice, allowing a broader investigation of sex‐specific cardiovascular responses in a genetically unmodified background. Preclinical studies commonly employ high‐salt diets (HSDs; 4%–8% NaCl) to induce cardiovascular changes within an experimentally feasible time frame.^12^, ^13^, ^14^, ^15^ By using 7‐ and 14‐month exposures, we capture both medium‐ and long‐term adaptations and provide insights into how chronic salt overload contributes to vascular stiffening, cardiac remodeling, and renal dysfunction.^6^ The herein described findings highlight the need to consider sex‐specific mechanisms when developing therapeutic interventions, extending beyond dietary salt reduction to more targeted and individualized approaches.

## METHODS

The authors declare that all supporting data are available within the article and its online supplementary files.

### Animals

All animal experiments presented in this study were approved by the institutional ethics committee at Lund University (#7143/2018) and were conducted in accordance with *Animal Research: Reporting of In Vivo Experiments* guidelines and European animal protection laws (Directive 2010/63/EU). Male and female 20‐ to 24‐week‐old C57BL/6JRj mice were purchased from Janvier Labs (Saint‐Berthevin, Le Geneste‐Saint‐Isle, France) and housed on a 12‐hour light–dark cycle in ventilated cages (Innovive, San Diego, CA, USA) with access to food and water ad libitum.

For this study, we selected wild‐type C57BL/6J mice to investigate sex‐specific cardiovascular responses to chronic high‐salt intake in a genetically unmodified background. Although typically considered salt resistant,^16^ C57BL/6J mice exhibit heterogeneity in blood pressure responses to HSD,^17^, ^18^ mirroring natural variations seen in humans, where salt sensitivity affects ~30% of healthy individuals and >50% of those with hypertension.^6^ This model allows for a more translationally relevant assessment of sex differences in salt handling and cardiovascular adaptation, without the confounding effects of preexisting genetic predispositions found in salt‐sensitive strains.

**Figure 1:**
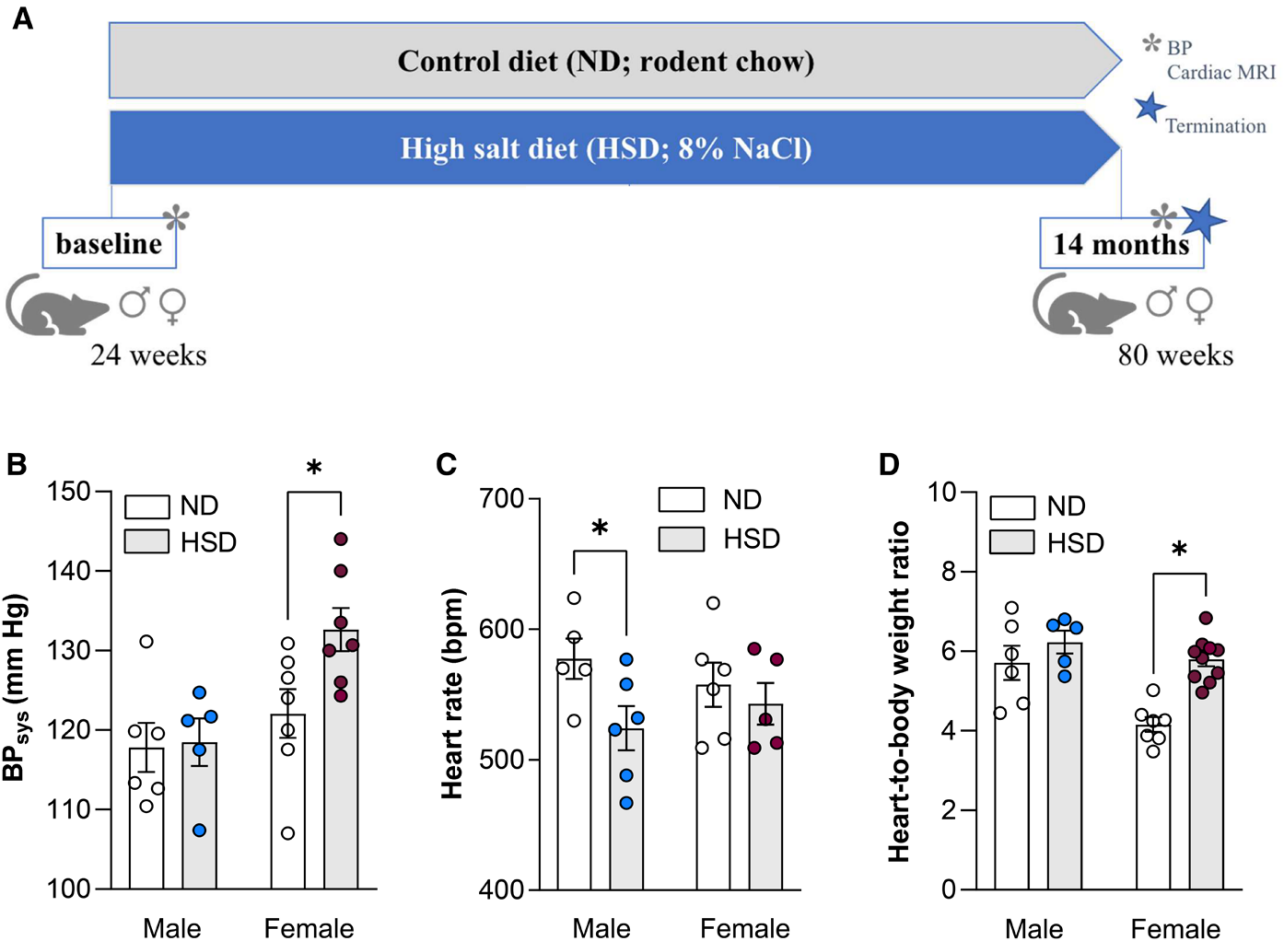
Chronic dietary exposure to high salt increases blood pressure in female but not male mice. **A**, Schematic representation of the experimental timeline. Tail cuff plethysmography was used to assess (**B**) systolic blood pressure and (**C**) heart rate in male and female mice on control diet and high salt diet. **D**, Organ‐to‐body weight ratio of the heart was calculated from values obtained at termination. Values are given as mean±SEM. *P<0.05 after 2‐way ANOVA and Fisher's least significant difference post hoc testing. BP_sys_ indicates systolic blood pressure; HSD, high‐salt diet; MRI, magnetic resonance imaging; and ND, normal diet.

At 28 weeks of age, mice were randomly assigned by cage to 1 of the following diet interventions for a total duration of 56 weeks (=14 months; Figure 1A): normal‐salt diet (ND) RM3‐P (Scanbur, Karlslunde, Denmark) containing 0.6% NaCl (n=12 males+15 females) or HSD consisting of chow diet with additional 8% (*w*/*w*) NaCl (n=13 males+13 females). A group of mice (n=9 males+10 females) were subjected to a diet reversal (REV) program that consisted of HSD feeding for 28 weeks (=7 months) followed by ND feeding for 28 weeks (=7 months). Food and water intake were recorded for 1 month after starting the diet intervention (ie, at 30 weeks of age) and at the time of diet reversal (ie, at 58 weeks of age).

### Blood Pressure Measurements

Blood pressure was measured in conscious mice using tail‐cuff plethysmography (CODA, Kent Scientific, Torrington, CT, USA) as previously described.^19^ Tail‐cuff plethysmography is a widely accepted noninvasive method for longitudinal blood pressure monitoring in rodents, which minimizes stress and adverse immune responses inherent to invasive methods, while allowing repeated measures over time.^20^ After a 1‐week handling period, mice were acclimatized to the restrainers and the tail cuff for a training period of 7 days. Data were recorded once mice presented with stable readings over the course of 1 week. We recorded 30 inflation cycles; the first 15 cycles were regarded as acclimatization and the remaining 15 were used for blood pressure analysis.

### Magnetic Resonance Imaging

Cardiac function was assessed using magnetic resonance imaging on a 9.4 T horizontal magnetic resonance scanner equipped with Bruker BioSpec AVIII electronics, a quadrature volume resonator coil (112/087) for transmission and a 20 mm linear surface loop coil for reception (Bruker, Ettlingen, Germany), operating with ParaVision 6.0.1. as previously described.^21^ During imaging, mice were immobilized using isoflurane (1.5%–2%) in room air, supplemented with 10% oxygen and kept at a respiration of 70 to 100 bpm and at 36 °C to 37 °C body temperature. Flow‐compensated fast low angle shot with ECG and respiration triggering (Stony Brook, NY, USA) with a resolution of 0.13×0.13×1 mm^3^ was used for all magnetic resonance scans. Positioning of the cardiac images was achieved by 3 orientational scans: (1) 3 axial slices (repetition time=50 ms, echo time=2.5 ms), (2) and (3) each with 1 slice (repetition time =6 ms, echo time=2.1 ms, 24 time frames) orthogonal to each other with slices positioned through the left and right ventricle and through the outflow tract of the left ventricle and the apex, respectively. Short‐axis view images of 9 to 10 slices (depending on heart size) were acquired with 24 time frames in each (repetition time =6 ms, echo time=2.1 ms). Hemodynamic parameters were assessed from the Dicom images using Segment (Medviso, Lund, Sweden).^22^ Relative wall mass was calculated as quotient of end‐diastolic LV mass (LVM) and end‐diastolic volume.

### Total RNA Isolation

Total RNA was isolated from homogenized left and right ventricles of the heart and from total kidney using TRIzol Reagent (#11312940, Thermo Fisher Scientific) according to manufacturer's instructions. RNA was extracted from mesenteric artery by using Stainless Steel Beads 5 mm Qiagen (#69989) to homogenize the tissue. 500 ng of RNA from heart and kidney and 150 ng of RNA from mesenteric artery was reversely transcribed to cDNA using High‐Capacity cDNA Reverse Transcription Kit (#4368814, Thermo Fisher Scientific). Gene expression was measured with quantitative reverse transcription polymerase chain reaction using Fast SYBR Green (#10209284, Applied Biosystems, Naerum, Denmark) and 0.2 μmol/L gene specific forward and reverse primers (Table S1) in a CFX384 Touch Real‐Time PCR Detection System (Bio‐Rad; Sundbyberg, Sweden). The polymerase chain reaction cycle parameters were 95 °C for 10 minutes, a total of 39 cycles (95 °C for 15 seconds, 60 °C for 1 minute) followed by a dissociation stage (95 °C for 1 minute, 55 °C for 70 seconds and 95 °C for 50 seconds). Data were analyzed using the absolute quantification method with standard curves generated from pooled cDNA representing each sample. Results were normalized to the housekeeping gene *Rpl14*; primer sequences are provided in Table S1.

### Immunohistochemistry

Following transcardiac perfusion with saline, tissues including aorta and kidney were collected and postfixed in 4% paraformaldehyde for 24 hours at 4 °C, cryoprotected in 30% of sucrose in PBS (in mmol/L: 137 NaCl, 2.7 KCl, 10 Na_2_HPO_4_, 1.8 KH_2_PO_4_) or paraffin‐embedded and stored at 4 °C or room temperature until sectioning.

Ten μm cryostat‐sectioned aorta cross sections were fitted onto SuperFrost Plus glass slides, washed with PBS and incubated for 30 minutes at room temperature with blocking buffer (#11096176001, Roche, Switzerland), followed by overnight incubation with primary antibodies against anti‐α‐SMA (alpha smooth muscle actin; #5228, Sigma Aldrich), anti‐OPN (osteopontin, AB10910, Sigma Aldrich) or anti‐CD68 (BioRad/Nordic Biosite Cat#MCA1957). After washing in PBS, samples were incubated with Alexa Fluor‐conjugated secondary antibodies (Goat Anti‐Rabbit Alexa Fluor 594 #A‐11037, Invitrogen, Goat Anti‐Mouse Alexa Fluor 488 #A‐11029, Invitrogen) for 1 hour, washed, mounted with Fluoromount‐G Mounting Medium with DAPI (#00–4959‐52, Invitrogen, USA), and examined under a BX60 (Olympus) and images were acquired with CellSens Dimension 1.5 Software. Three to 4 histological sections per specimen were evaluated using the TissueQuant image analysis tool^23^ and ImageJ (https://imagej.net; version.1.52i) for SMA, elastin, CD68, and OPN quantification. Representative images were visualized using a 40× objective and processed in ImageJ.

Five μm microtome‐sectioned kidney sections were fitted onto SuperFrost glass slides and placed at 60 °C for 1 hour to melt the paraffin. After deparaffinization with xylene and rehydration with 100%, 90%, and 70% ethanol, slides were washed in PBS, supplemented with 0.25% Triton‐X‐100. Nonspecific binding was blocked using 2% serum in PBS for 1 hour at room temperature, followed by an overnight incubation with primary rabbit anti‐CD45 antibody (1:500, Biolegend, 103102). After washing in PBS, samples were incubated with Alexa Fluor‐conjugated goat anti‐rabbit secondary antibody for 1 hour at room temperature. Following washing with PBS, slides were mounted with Prolong Gold Antifade Reagent with DAPI (#8961) and examined under a BX60 (Olympus) and images were acquired with CellSens Dimension 1.5 Software. A minimum of 10 glomeruli per animal was examined and an average of cells/glomeruli is reported.

### Histology

Ten μm cryostat‐sectioned aorta or 5 μm microtome‐sectioned kidney cross sections were and subjected to hematoxylin‐eosin, Masson trichrome, and Van Gieson staining.

For hematoxylin‐eosin staining, aorta sections were washed by PBS and incubated in Meyers hematoxylin (Histolab) for 10 minutes, HCl‐ethanol (0.3% in 70% ethanol) for 1 minute, tap water for 5 minutes, and eosin (0.5 g in 70% ethanol) for 1 minute. After dehydration, glass slides were covered with coverslips using Rotimount (Carl Roth) and imaged with an Olympus BX60 using the CellSens Dimension 1.5 Software (Olympus). For quantification of aortic vessel wall thickness, at least 5 vessel sections per animal were manually assessed using the “straight line” tool in ImageJ in scale‐adjusted images.

Kidney sections were deparaffinized using xylene and rehydrated using 100%, 95%, and 70% ethanol for 4 minutes each. Kidney sections were incubated with hematoxylin for 5 minutes, HCl‐ethanol (0.3% in 70% ethanol) for 1 minute, tap water for 5 minutes, followed by a 3‐minute incubation with eosin. After rinsing the sections with deionized water, the sections were dehydrated using ascending ethanol steps and xylene for 1 minute each before the sections were mounted using Rotimount and imaged with a Nikon Ti2e microscope using Nikon Instruments Software elements software. For quantification of kidney vessel wall thickness, a minimum of 8 vessels per animal were manually assessed using the “straight line” tool in ImageJ in scale‐adjusted images. Analyzed vessels were classified based on vessel diameter. Bowman capsule area and tuft area were assessed using the Freehand selection and Measure tools in Image J. Ten glomeruli per animal were analyzed. For semiquantitative scoring of glomerular damage, we used the quantification tool in the NetScope Viewer software. At a fixed 5× magnification, the tool divides the entire image into equal‐sized grids. We chose glomeruli lying within every other grid and assigned a score based on the extent and type of observed damage, ranging from 0 to 4. Healthy glomeruli showing no structural aberrations were scored 0. Glomeruli showing podocyte hypertrophy and mild mesangial expansion were scored 1. Glomeruli displaying mesangial matrix expansion and <50% sclerosis were scored 2. Glomeruli exhibiting >50% sclerosis were scored 3. Glomeruli demonstrating global sclerosis were deemed nonfunctional and assigned score 4. Incidence of protein casts and interstitial tubular fibrosis was visualized in pie‐donut‐plots (code: see Data S1).

For Masson trichrome staining, a commercially available kit (#HT15, Sigma Aldrich) was used as per manufacturer's instructions with minor adjustments. Briefly, sections were fixed in Bouin's solution (75% saturated picric acid, 10% formaldehyde, 5% glacial acetic acid) for 5 minutes at 57 °C then washed under running tap water for 3 minutes and rinsed in H_2_O. Sections were further stained with Weigert's iron hematoxylin for 1 minute and subsequently washed under running tap water for 5 minutes before rinsing in H_2_O and staining with Biebrich scarlet‐acid fuchsin for 45 seconds. Sections were incubated with phosphotungstic/phosphomoloybdic acid for 3 minutes before placing them aniline blue solution for 45 seconds followed by a 1‐minute incubation in 1% acetic acid. Glass slides were covered with coverslips using Rotimount and imaged with an Olympus BX60 or an Olympus CX33 using the CellSens Dimension 1.5 Software (Olympus). For the qualitative quantification of collagen in Masson trichrome stained aortas staining intensity was determined with ImageJ by setting the color deconvolution plugin for Masson trichrome as previously reported.^24^ This plugin allows for the separation of colors via color deconvolution, which provides separation of collagen fibers from the overlapping regions. Five sections per animal were evaluated. For the kidneys, a minimum of 5 microscopic fields of glomeruli was evaluated per animal.

Van Gieson staining was performed as per manufacturer's instructions. Briefly, sections were incubated in Van Gieson solution (#HT254, Sigma‐Aldrich) for 1.5 minutes and rinsed 2 times in 95% alcohol and twice in absolute alcohol for 2 minutes each before mounting with coverslips using Rotimount (Carl Roth). The sections were visualized with a DMI6000B microscope (Leica, Wetzlar, Germany), and images were acquired with Leica LAS X. ImageJ with color deconvolution plugin was used for qualitative quantification of collagen from Van Gieson staining.

### Plasma Nitrite Levels

Plasma nitrite levels were measured as an indicator of nitric oxide availability using Griess assay (#EMSNOTOT, Thermo Fisher) as per the manufacturer's instructions.

### Wire Myography

Mesenteric arteries (second order, 150–200 μm) were dissected using a stereoscope (Nikon SMZ745T) and mounted using steel wire in myograph chambers (610M and 620M, Danish Myo Technology) containing HEPES‐buffered Krebs solution (in mmol/L: 135.5 NaCl, 5.9 KCl, 2.5 CaCl_2_, 1.2 MgCl_2_, 11.6 glucose, and 11.6 HEPES, pH 7.4).^25^ After heating to 37 °C, segments were slowly stretched to a basal tension of 5 mN, and equilibrated for 30 minutes before artery viability was tested using 60 mmol/L KCl. Following 3 washes with HEPES‐buffered Krebs solution and 30 minutes equilibration at 37 °C, dose response curves to alpha‐1 adrenergic stimulation using the selective alpha‐1 adrenoreceptor agonist cirazoline were generated (#223, Sigma Aldrich).^26^ Artery relaxation was determined through acquisition of dose‐response curves to the parasympathomimetic carbachol (#C4382, Sigma Aldrich) after alpha‐1 adrenergic activation using 0.3 μmol/L cirazoline or 60 mmol/L KCl. Artery relaxation is expressed as force reduction from the force of the precontracted artery set as 100%. Vessel force was continuously monitored using LabChart software (ADInstruments).

### Statistical Analysis

Using previous data as guidance,^27^ the experimental group sizes necessary to ensure that all data provide a power of 80% power (1‐β >0.8) and a 2‐tailed Type I alpha error of 0.05 was calculated. All assessments and analyses in the current study were performed under blinded conditions, using codes that concealed the identity of the intervention. Results are presented as mean±SEM unless otherwise stated and were analyzed with Prism 10.2.3 (GraphPad, San Diego, CA, USA). All data sets were tested for Gaussian distribution using Shapiro–Wilk normality test. Two‐way ANOVA or repeated measures 2‐way ANOVA followed by Fisher's least significant difference post hoc testing was used to test differences between multiple groups defined by 2 factors. In repeated measures 2‐way ANOVA models, treatment group (diet), sex, and their interaction were treated as fixed effects, whereas subject ID was treated as a random effect to account for intrasubject correlation over time. To test the effect of vascular responses to vasoactive substance, diet or sex, drug concentration, and their interaction were treated as fixed effects. Similarly, diet or sex, time, and their interaction were treated as fixed effects when longitudinally assessing effects on blood pressure and cardiac function parameters. The covariance structure was modeled as compound symmetry, as implemented in GraphPad Prism version 10.2.3 and later versions. For all data sets, N represents the number of animals. Differences were considered significant at *P*<0.05. Additional statistical details are provided in Table S2.

## RESULTS

### Chronic Dietary Exposure to High Salt Increases Blood Pressure in Female But Not Male Mice

Starting at 28 weeks of age, mice were subjected to an ND diet (0.6% [*w*/*w*] NaCl) or an HSD consisting of a chow diet with an additional 8% (*w*/*w*) NaCl for 56 consecutive weeks (Figure 1A). Relative to the ND group, mice subjected to HSD showed reduced weight gain despite increased average food and water intake (Figure S1A through S1D). After 56 weeks of HSD, significantly higher systolic blood pressure levels were recorded in female but not in male mice (Figure 1B). Notably, heart rate was significantly reduced in male HSD mice but remained unchanged in females (Figure 1C). Longitudinal assessment revealed a steady decline in heart rate in all experimental groups over time and a significant lowering in male mice after 14 months of HSD exposure (Figure S2A and S2B). Organ‐to‐body weight ratio of the heart was higher in female HSD mice but not in male counterparts (Figure 1D).

### Chronic Dietary Exposure to High Salt Is Associated With Left Ventricular Alterations in Male But Not Female Mice

**Figure 2:**
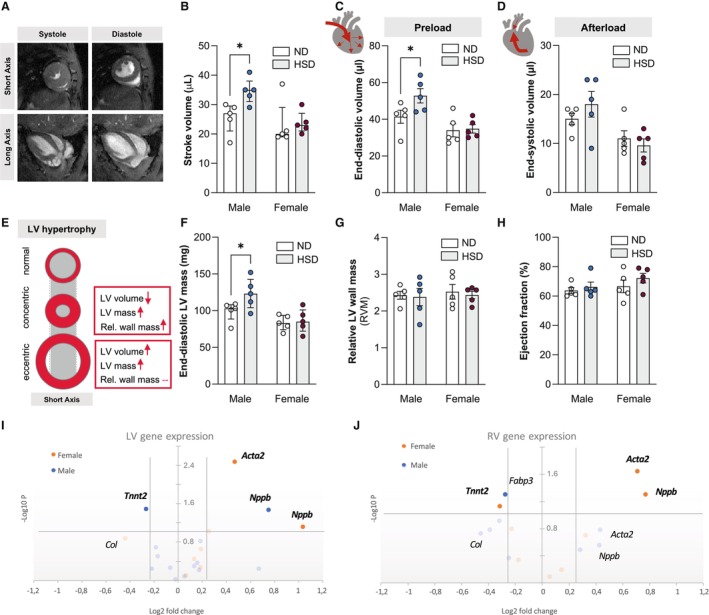
Chronic dietary exposure to high salt increases associates with left ventricular alterations in male but not female mice. MRI was used to assess cardiac function. **A**, Representative images showing short‐ and long‐axis views in systole and diastole. MRI‐based analyses showing (**B**) increased stroke volume and (**C**) end‐diastolic volume in male mice on HSD compared with ND, and (**D**) no differences in end‐systolic volume between diets. **E**, Schematic showing characteristics of different types of LV hypertrophy. MRI‐based analyses revealing (**F**) increased end‐diastolic LV mass in male HSD mice but (**G**) unaltered relative LV wall mass and (**H**) LV ejection fraction between the groups. Diet‐dependent gene expression in (**I**) left and (**J**) right ventricle showing sex‐dependent differences. Each circle represents 1 gene. The log fold changes vs the respective control groups are represented on the *x* axis. Gene names are given for targets with most apparent changes after 56 wk of HSD. Significant targets in bold. Dashed lines show cutoffs of fold‐change=±0.25, and of *P*=0.05. In (**B**) and (**F**), values are given as median±interquartile range; in (**C**), (**D**), (**G**), and (**H**), values are given as mean±SEM. *P<0.05 after two‐way ANOVA and Fisher's least significant difference post hoc testing. HSD indicates high‐salt diet; LV, left ventricular; MRI, magnetic resonance imaging; ND, normal diet; and RV, right ventricular.

Despite an apparent hypertensive phenotype in HSD‐fed female mice, cardiac function was impaired only in HSD‐fed male mice after 56 weeks of excessive salt intake. Magnetic resonance imaging‐based cardiac function assessment (Figure 2A) revealed significantly higher stroke and end‐diastolic volumes (Figure 2B and 2C) but not end‐systolic volume (Figure 2D) in male but not in female mice. Longitudinal monitoring showed increasing stroke and end‐diastolic volumes over the course of HSD feeding only in male HSD mice (Figure S2C and S2D). Changes in LV geometry, such as increases in LVM accompanied by wall thickening or LV cavity enlargement, are common adaptive responses to prolonged pressure or volume overload (Figure 2E).^28^, ^29^ In response to chronic salt loading, higher end‐diastolic volume and LVM (Figure 2F) resulted in an unchanged relative LVM‐to‐volume ratio (Figure 2G) in male mice, suggesting eccentric LV hypertrophy in response to HSD in male mice.^30^ Ejection fraction remained unaltered by HSD in both male and female mice (Figure 2H). Expression profiling of cardiac injury markers revealed diet effects for LV *Nppb* (Figure 2I). Specifically, male HSD‐fed mice presented with significantly lower LV *Tnnt2* expression compared with ND controls, whereas female HSD mice showed higher *Acta2* levels compared with their respective ND controls (Figure 2I; Table S3). Similarly, diet effects were detected in the right ventricle for *Nppb*, *Tnnt2*, and *Acta2* expression in female mice and for *Fabp3* expression in male mice (Figure 2J; Table S4).

### Chronic Dietary Exposure to High Salt Alters Conduit and Resistance Vessel Structure and Function

**Figure 3:**
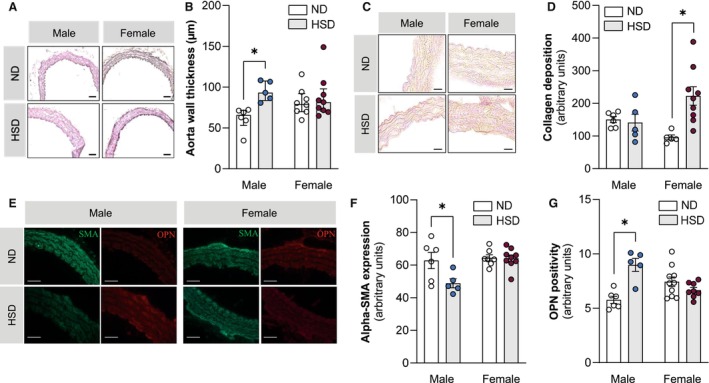
High‐salt diet alters aortic cell content and morphology. **A**, Representative images of aortas from ND and HSD male and female mice stained with H&E. Scale bar: 100 μm. **B**, Quantification of H&E staining showing increased aortic wall thickness in male HSD mice. **C**, Representative images of VG aortas from ND and HSD male and female mice. Scale bar: 20 μm. **D**, Quantification of VG staining showing higher aortic collagen deposition in female compared with male HSD mice. **E**, Representative images of aortas from ND and HSD male and female mice stained with SMA (green) and OPN (red). Scale bar: 20 μm. Quantification showing (**F**) less SMA^+^ and (**G**) more OPN^+^ cells in male HSD mice. In (**B**), values are given as median±interquartile range; in (**D**), (**F**), and (**G**), values are given as mean±SEM. *P<0.05 after 2‐way ANOVA and Fisher's least significant difference post hoc testing. H&E indicates hematoxylin‐eosin; HSD, high‐salt diet; OPN, osteopontin; SMA, smooth muscle actin; and VG, Van Gieson.

Aorta wall thickness was higher in male but not female mice after 56 weeks of HSD feeding (Figure 3A and 3B). Despite this, aorta collagen deposition determined by Van Gieson staining showed a statistically significant increase with HSD in female but not male mice (Figure 3C and 3D). Moreover, aortas from male HSD‐fed mice showed less SMA positivity as compared with ND controls (Figure 3E and 3F). This was accompanied by less nuclei per SMA^+^ area (Figure S3A), suggestive of a lower number of smooth muscle cells, which is common in aging and hypertension.^31^, ^32^ Interestingly, higher OPN positivity in male HSD mice (Figure 3E and 3G) results in significantly lower SMA:OPN ratios (Figure S3B), suggestive of synthetic phenotype predominance.^33^, ^34^, ^35^ In contrast to OPN, CD68 positivity, which is generally indicative of vascular remodeling and inflammation,^36^ was unaffected by HSD (Figure S3C).

**Figure 4:**
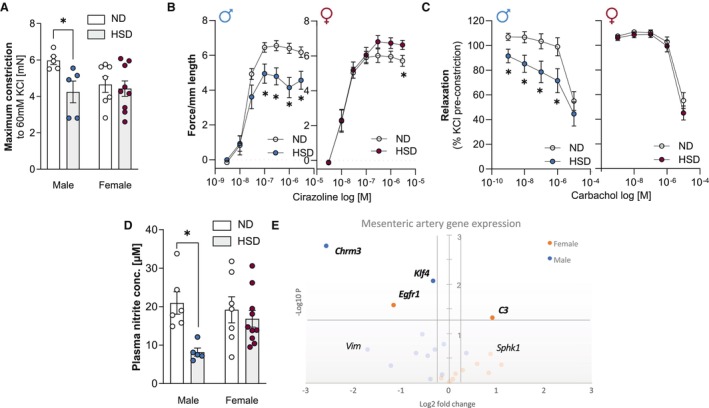
Chronic dietary exposure to high salt alters vascular responsiveness and gene expression. Wire myography used to assess vascular functionality showing (**A**) lower maximum constriction to KCl (60 mmol/L) in male mice on HSD compared with ND. Vessel tension in response to increasing concentrations of (**B**) the alpha 1 adrenergic receptor agonist cirazoline was lower in male and higher in female HSD mice compared with their respective ND controls. **C**, Vessel relaxation in response to increasing doses of the parasympathomimetic carbachol after KCl preconstriction was impaired in male but not female HSD mice compared with their respective ND controls. **D**, Griess assay showing reduced plasma nitrite concentrations in male, but not female HSD mice. **E**, Diet‐dependent gene expression in mesenteric artery extracts showing sex‐dependent differences. Each circle represents 1 gene. The log fold‐changes vs the respective control groups are represented on the *x* axis. Gene names are given for targets with most apparent changes after 56 wk of HSD. Significant targets in bold. Dashed lines show cutoffs of fold‐change=±0.25, and of *P*=0.05. Values are given as mean±SEM. In (**A**) and (**D**), *P<0.05 after 2‐way ANOVA and Fisher's LSD post hoc testing. In (**B**) and (**C**), *P<0.05 after RM‐ANOVA and Fisher's LSD post hoc testing. HSD indicates high‐salt diet; LSD, least significant difference; and ND, normal diet.

Mesenteric arteries are important contributors to total peripheral resistance^37^ and therefore, to blood pressure alterations during the development and progression of hypertensive disease.^19^, ^38^, ^39^ After 56 weeks of HSD in male, but not female mice, mesenteric arteries presented with a reduced ability to constrict in response to potassium chloride or alpha‐1 adrenergic stimulation (Figure 4A; Figure S4A). Moreover, alpha‐1 adrenergic activation yielded significantly higher vessel tension in HSD mice compared with ND‐fed controls in females, whereas male HSD mice showed a significantly lower response to different concentrations of cirazoline, a selective alpha‐1 adrenergic agonist (Figure 4B). Dose‐dependent contraction responses to cirazoline were lower in male than in female HSD mice (Figure S4B). Mesenteric artery relaxation in response to the parasympathomimetic carbachol after potassium chloride preconstriction was significantly lower in male HSD‐fed mice compared with ND controls, whereas female groups did not differ (Figure 4C). Likewise, male HSD mice presented with significantly lower vasodilatory responses compared with female HSD mice (Figure S4C). Relaxation after cirazoline preconstriction was not significantly different between the groups (Figure S4D through S4F). In line with these functional differences, lower plasma nitrite concentrations, measured as an indicator of nitric oxide availability, were detected in HSD‐fed male but not female mice compared with their respective controls (Figure 4D). Gene expression analyses of mesenteric arteries confirmed sex‐specific differences in response to HSD (Figure 4E; Table S5). Here, significant diet effects were detected for *Egr1* and complement C3 (*C3*) in female mice, while HSD affected muscarinic receptor 3 (*Chrm3*) and *Klf4* in male mice (Figure 4E; Table S5). Interestingly, expression of the majority of tested genes are augmented in response to high dietary salt intake in female and are reduced or not affected in male mice.

### Renal Marker Expression Is Altered by Chronic High Salt Intake

**Figure 5:**
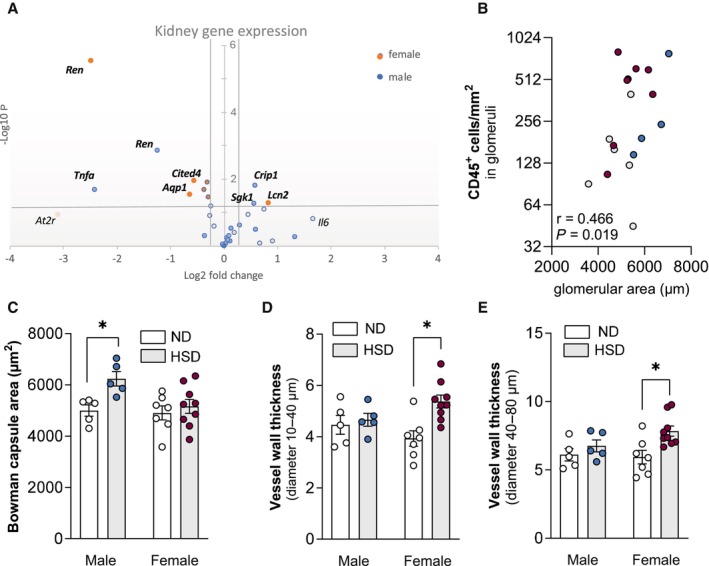
Kidney effects of exposure to high dietary salt. **A**, Diet‐dependent kidney gene expression showing sex‐dependent differences. Each circle represents 1 gene. The log fold‐changes vs the respective control groups are represented on the *x* axis. Gene names are given for targets with significant changes after 56 wk of HSD. Significant targets in bold. Dashed lines show cutoffs of fold‐change=±0.25, and of *P*=0.05. **B**, Spearman correlation showing a positive association between the number of infiltrated CD45^+^ cells and Bowman capsule size. **C**, Enlargement of the Bowman capsule area in male HSD mice. In female but not male mice, renal vessel thickness is enlarged in vessels with diameters between (**D**) 10 to 40 μm and (**E**) 40 to 80 μm. Values are given as mean±SEM. *P<0.05 after 2‐way ANOVA and Fisher's least significant difference post hoc testing. HSD indicates high‐salt diet; and ND, normal diet.

Renal markers of water homeostasis, renin‐angiotensin‐aldosterone system alteration as well as kidney inflammation have been used to assess renal involvement in hypertension and associated kidney injury.^40^, ^41^ Chronic high salt intake lowered renal renin gene expression in both sexes and altered expression of aquaporins relevant for aldosterone‐dependent and ‐independent water absorption^42^, ^43^ in female HSD‐fed mice (Figure 5A; Table S6). Expression levels of *Crip1* and *Sgk1* were augmented in kidney tissue of male HSD‐fed mice (Figure 5A; Table S6). In kidneys from female HSD mice, expression of *Aqp1* and *Agt1r* was lowered (Figure 5A; Table S6), suggestive of alterations of water homeostasis and inflammatory responses.^44^, ^45^ In line with higher expression of inflammation‐related genes, the number of CD45^+^ cells infiltrated into glomeruli positively associated with increasing Bowman capsule area (Figure 5B). When stratifying by sex, the number of infiltrated CD45^+^ cells was highest in kidneys of female HSD mice (342±149 versus 87±66 in males and 413±89 versus 70±30 in females). Enlargement of Bowman capsule area in response to HSD was most apparent in male mice (Figure 5C). On the contrary, wall thickness of cortical vessels with diameters between 10 and 40 or 40 and 80 μm augmented with HSD‐feeding in female mice only (Figure 5D and 5E).

### Lowering Dietary Salt Content Improves Blood Pressure, Cardiac Function, and Kidney Parameters

**Figure 6:**
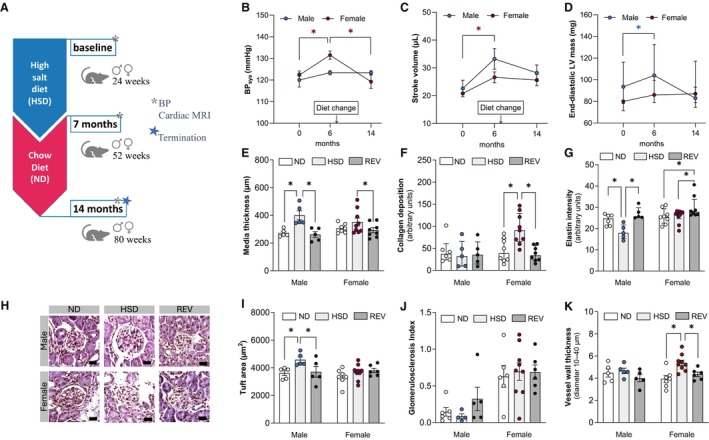
Lowering dietary salt content improves cardiac function, blood pressure, and kidney parameters. **A**, Schematic showing experimental timeline. Lowering dietary salt content (**B**) reduces elevated systolic blood pressure in female mice and (**C**) lowers elevated left ventricular stroke volume and (**D**) end‐diastolic left ventricular mass in male mice. **E**, Aorta media thickness is highest in male HSD mice. **F**, Masson trichrome stained aortas showing higher collagen accumulation in the HSD but not after diet reversal in female mice. **G**, Aorta elastin content is lowest in male HSD mice. **H**, Representative images showing hematoxylin–eosin‐stained Bowman capsules in kidney sections from male and female mice on ND, HSD, and REV. Scale bar: 20 μm. **I**, Tuft area enlargement in male HSD mice is not observed in male mice after diet reversal. **J**, HSD feeding or subsequent diet reversal have no effect on the glomerular health of male and female mice. **K**, Mice after REV did not show alterations in renal vessel wall thickness in vessels with diameters between 10 and 40 μm. In (**B**), (**C**), (**E**), (**H**), (**I**), and (**K**), values are given as mean±SEM; in (**D**), (**F**), and (**G**), values are given as median±interquartile range. **B** through **D**, *P<0.05 after repeated measure ANOVA and Fisher's LSD post hoc testing. In (**E**), (**F**), (**G**), (**H**), (**I**), and (**K**), *P<0.05 after 2‐way ANOVA and Fisher's LSD post hoc testing. BP_sys_ indicates systolic blood pressure; HSD, high‐salt diet; LSD, least significant difference; LV, left ventricular; MRI, magnetic resonance imaging; ND, normal diet; and REV, diet reversal.

In a separate group of mice, HSD diet was reversed to ND after 28 weeks of feeding (Figure 6A). Systolic and mean arterial blood pressures that increased with HSD feeding in female mice were lowered to baseline levels during 28 weeks after diet reversal (Figure 6B; Figure S5A). Heart rate nonsignificantly lowered over the course of the experiment, showing no apparent effects of diet reversal (Figure S5B). Cardiac effects of chronic exposure to high salt, including elevated stroke volume and end‐diastolic LVM in male mice were lowered by diet reversal (Figure 6C and 6D), whereas end‐diastolic volume remained unchanged (Figure S5C).

As age notably affects cardiovascular structure and function, invasive assessments of blood vessels and kidney parameters were performed at the 56‐week study time point, and the REV group was compared with HSD and ND groups. Aorta wall and media thickness was not increased in the REV group (Figure S6A, Figure 6E). Aorta collagen deposition as determined by Masson trichrome staining confirmed a statistically significant increase with HSD in female mice; this effect was absent in the REV group (Figure 6F). Elastin intensity was lower in the HSD compared with ND group in male mice only; this was not observed in the REV group (Figure 6G). Similarly, aortic SMA expression was lower in male REV mice compared with age‐matched ND controls (Figure S6B), whereas OPN expression was higher (Figure S6C). The resulting SMA:OPN ratios were significantly lower in male HSD and REV groups compared with ND controls (Figure S6D).

Similarly, renal marker responses were differently affected by diet reversal. Renin expression was significantly higher in REV compared with HSD mice independent of sex (Figure S7A). The HSD‐associated alterations of renal *Aqp‐2* and *Cd86* expression in female mice were not detected in the REV group (Figure S7B and S7C). *Lcn2* expression that elevated with HSD feeding in female mice was further augmented with diet reversal (Figure S7D).

Histologically, HSD feeding led to pronounced glomerular hypertrophy as evidenced by tuft area enlargement (Figure 6H and 6I). To further examine whether HSD feeding accelerates glomerulosclerosis, a process also commonly linked to aging, we employed a semiquantitative scoring of various glomerular damages. This approach assesses several histological features, including podocyte hypertrophy, mesangial matrix expansion, and progressive scarring leading to glomerular fibrosis. Representative images for each score illustrating our scoring framework with distinct glomerular damages highlighted are provided in Figure S8A. The overall glomerulosclerosis index remained unaffected by the dietary regimen in both male and female mice (Figure 6J). However, female mice, including the ND controls, exhibited higher glomerulosclerosis indices compared with their male counterparts, suggesting a trend toward greater susceptibility in females. This increased propensity for renal injuries in female animals is further emphasized by the higher incidence of protein casts (Figure S8B through S8D). Although no abnormal deposition of protein in the tubular lumen was observed in male mice fed a HSD, 33% of male mice in the ND group exhibited age‐related protein cast deposition, and 20% of male mice in the REV group showed protein casts (Figure S8C). Among female mice, all animals of HSD and REV groups showed protein casts, compared with 86% of ND‐fed mice (Figure S8D). Furthermore, all male mice in the HSD and ND groups exhibited mild tubular interstitial fibrosis (representative image displayed in Figure S8E), whereas 60% of male mice in the REV group showed fibrosis (Figure S8F). In female mice, tubular interstitial fibrosis was observed in 43% of the ND group, 89% of the HSD group, and 71% of the REV group (Figure S8G). Additionally, vessel wall thickening in the renal cortex was also observed in the REV group in vessels with diameters between 10 and 40 μm (Figure 6K) but not in vessels with diameters between 40 and 80 μm (Figure S9).

## DISCUSSION

The present study demonstrates that chronic high salt intake induces pronounced sex‐specific cardiovascular adaptations, with female mice developing salt‐sensitive hypertension and male mice exhibiting significant cardiac and vascular remodeling without concurrent increases in blood pressure. These findings are consistent with the growing body of evidence suggesting that women exhibit heightened salt sensitivity of blood pressure compared with men.^5^, ^7^

In contrast to females, male mice did not exhibit significant increases in blood pressure following chronic high salt intake. This resistance to salt‐induced hypertension may be attributed to greater renal sodium excretion, which has been reported in male subjects compared with female subjects,^46^, ^47^ as well as differential sodium handling mechanisms, vascular adaptations or autonomic adjustments that counteract the pressor effects of high salt. Male HSD mice displayed reduced vascular contractile responses to adrenergic stimulation and lower expression levels of muscarinic receptors, consistent with alterations in autonomic balance. Notably, previous studies have reported increased vagal tone in response to high salt exposure,^48^, ^49^ which may contribute to the observed heart rate reduction and vascular adaptations. Over the course of high‐salt feeding, end‐diastolic volume and LVM increased in male mice without apparent changes in ejection fraction, a pattern indicative of eccentric hypertrophy.^50^ This together with the observed increases in left ventricular stroke volume are suggestive of a compensatory response rather than systolic dysfunction. Despite the changes in stroke volume, lower *Tnnt2* expression in the LV, reflecting reduced troponin T availability,^51^ may predispose to impairment of myocardial contractility long term.^52^, ^53^ Assessment of cardiac function at later time points would inform about a potential transition to systolic dysfunction. Importantly, because indicators of increased peripheral resistance were observed only in female HSD mice, the cardiac remodeling in males is unlikely to be due to pressure overload. Instead, these findings suggest that male hearts are susceptible to structural remodeling even in the absence of overt hypertension, a phenomenon documented in other models of salt‐sensitive cardiovascular disease.^54^, ^55^, ^56^ Blood pressure increases in female HSD mice on the other hand are primarily driven by vascular changes rather than cardiac adaptations as vascular remodeling in response to high salt intake displayed clear sex‐specific differences. Specifically in the mesenteric arteries, which play a pivotal role in regulating total peripheral resistance,^57^ sex‐based differences in vascular function were also discernible. Although male mice showed impaired contractile responses to both potassium chloride and alpha‐1 adrenergic stimulation, female mice responded with augmented contraction. Downregulation of the alpha‐1 adrenergic receptor, which mediates direct contractile responses in mesenteric arteries^26^, ^58^, ^59^ together with the elevation of *Klf4*, which inhibits the expression of contractile marker genes in vascular smooth muscle cells^60^ in male HSD mice are indicative of reduced vascular reactivity and responsiveness to adrenergic stimulation, whereas enhanced contractile response observed in females may contribute to the development of hypertension by increasing peripheral resistance.^61^ Moreover, extracellular matrix remodeling in female HSD mice as evidenced by increased collagen deposition in the aorta may contribute to vascular stiffening, reduced vascular compliance^62^ and elevated pulse pressure that hence augment systolic blood pressure.^57^ These observations are in stark contrast to those observed in male HSD mice where increased aortic wall thickness and reduced SMA expression together with elevated OPN expression suggest a transition to a synthetic smooth muscle cell phenotype^33^, ^34^, ^63^ with diminished contractility and augmented extracellular matrix synthesis.^33^, ^64^ These results collectively indicate that males and females use disparate mechanisms of cardiovascular adaptation in response to chronic salt intake. Although the hypertensive response to HSD in females is likely driven by vascular stiffening and increased vascular reactivity rather than cardiac remodeling or changes in contractility, male HSD mice may undergo autonomic adaptation, leading to cardiac remodeling.

The kidney is central to blood pressure regulation,^65^, ^66^ and our results suggest mild sex‐specific differences in renal responses to high salt intake. Both male and female mice exhibited reduced renal renin expression, consistent with previous studies showing downregulation of the renin‐angiotensin‐aldosterone system in response to high salt.^67^ Although in female mice long‐term exposure to high salt altered the expression of aquaporins relevant for aldosterone‐dependent and ‐independent water absorption,^42^, ^43^ male HSD mice presented with augmented *Crip1* expression, whose plasma levels are strongly associated with increases in blood pressure and cardiac hypertrophy,^68^ as well as higher *Sgk1* expression levels, which have been suggested as a risk factor for the development of mineralocorticoid‐dependent kidney injury independently of blood pressure.^69^ These findings indicate the presence of early renal damage and suggest that males may be more susceptible to salt‐induced kidney injury, despite their relative protection from hypertension. In contrast, female mice exhibited alterations in aquaporin expression, which are involved in water reabsorption,^70^ suggesting that water handling mechanisms may be differentially affected by high salt intake in females. Nevertheless, the early gene expression changes observed did not translate to significant histological alterations beyond the typical age‐related pathology, despite long term HSD exposure.

Importantly, our results demonstrate that reducing dietary salt intake after prolonged high salt exposure can mitigate many of the adverse cardiovascular and early renal effects. Following a 28‐week dietary salt reduction, improvements were primarily observed in blood pressure and cardiac function. These findings highlight the potential for dietary interventions to reverse salt‐induced cardiovascular damage. However, they also underscore the importance of early intervention to prevent irreversible remodeling.

### Study limitations

We recognize there are limitations with the current study. Although the use of wild‐type C57BL/6J mice allows the study of naturally occurring sex‐specific responses to high salt intake, these mice are generally considered to be salt resistant,^16^ which may underestimate the full spectrum of salt‐sensitive hypertension observed in certain human populations. Blood pressure was measured by tail‐cuff plethysmography, a widely used method for longitudinal systolic blood pressure monitoring,^20^ but which lacks the reliable capability to determine diastolic blood pressure levels. In addition, age‐related cardiovascular changes may contribute to some findings. However, because all experimental groups aged concurrently, any age‐related effects would be expected to similarly influence all groups, making diet and sex the primary variables driving observed differences. Finally, although we comprehensively examined cardiac and vascular remodeling, future studies incorporating molecular and cellular analyses of renal function may provide further mechanistic insights into sex differences in salt handling and cardiovascular adaptation.

### Conclusions

Chronic high salt intake induces distinct cardiovascular and renal adaptations in male and female mice, with females developing salt‐sensitive hypertension and males exhibiting significant cardiac and vascular remodeling. These sex‐specific responses underscore the need for personalized approaches to managing salt‐sensitive hypertension and highlight the importance of considering sex differences in both basic and clinical research. Further studies are needed to elucidate the underlying mechanisms driving these differences and to develop targeted therapeutic strategies for salt‐sensitive cardiovascular disease.

## Sources of Funding

This work was funded by the Anna‐Lisa Rosenberg Foundation, the Swedish Research Council (2019‐01130, João M. N. Duarte; 2022‐00973, Martin Magnusson, Anja Meissner, and João M. N. Duarte), Direktör Albert Påhlssons stiftelse (João M. N. Duarte; Anja Meissner) and the Swedish Heart and Lung Foundation (2024‐ 0979, Martin Magnusson). The Knut and Alice Wallenberg foundation, the Faculties of Medicine at Lund University and the University of Augsburg, and Region Skåne are acknowledged for generous financial support to Kerstin Stemmer, João M. N. Duarte, Anja Meissner, and Martin Magnusson. The authors acknowledge support from the Lund University Diabetes Center, which is funded by the Swedish Research Council (Strategic Research Area EXODIAB, grant 2009‐1039) and the Swedish Foundation for Strategic Research (grant IRC15‐0067). Lund University Bioimaging Centre (LBIC) is gratefully acknowledged for providing experimental resources.

## Disclosures

The authors have no conflicts of interest to declare.

## Supporting information

Data S1
Tables S1–S6
Figures S1–S9
