# Supplementary material for: Sex‐Specific Cardiovascular Consequences of Long‐Term High‐Salt Diet in Mice
Source: J Am Heart Assoc. 2025 Oct 10;14(20):e041945. doi: 10.1161/JAHA.125.041945 (PMC12684615; doi:10.1161/JAHA.125.041945)
Supplement: Supplementary file 1 — Data S1 Tables S1–S6 Figures S1–S9 [file JAH3-14-e041945-s001.pdf]

SUPPLEMENTAL MATERIAL

Data S1. Code to generate PieDonut plots.

```
# Install and load necessary packages
install.packages("openxlsx")
library(openxlsx)
if(!require(devtools)) install.packages("devtools")
devtools::install_github("cardiomoon/moonBook")
devtools::install_github("cardiomoon/webr")
require(ggplot2)
require(moonBook)
require(webr)
library(webr)
if (!require(dplyr)) install.packages("dplyr")
library(dplyr)
if (!require(tidyr)) install.packages("tidyr")
library(tidyr)
#set working directory containing the raw data file
setwd("/Users/SnehaPrakash/Desktop/Anja")
sheetname <- c("Fibrosis Female MT") #Insert the name of the excel sheet containing the raw data for the dataframe
plot_title <- c("Presence of fibrosis in Female mice: MT") #Your title for the final graph
plot_name <- c("Fibrosis in Female MT.png")
Incidencedf <- read.xlsx("Sneha_HSD kidney analysis_Final.xlsx",
                         sheet = sheetname) #Read raw data from the excel sheet and store in a dataframe
Incidencedf # View & confirm the raw data
# Create and export the PieDonut plot for protein casts incidence
png(plot_name, res = 300, width = 1000, height = 1000)
par(mar = c(4,4,1,1))
PieDonut(
  Incidencedf,
  aes(pies = Treatment_group, donuts = fibrosis_presence, count = Count), # change to "donuts = Protein_cast_presence" for protein casts incidence
  labelposition = 1,
```

```
  showPieName = FALSE,
  showRatioPie = FALSE,
  donutLabelSize = 3,
  pieLabelSize = 3,
  showRatioThreshold = 0.00,
  r0 = 0.2,
  r1 = 0.5,
  r2 = 0.6,
  title = plot_title,
  titlesize = 4
)
dev.off()
?PieDonut #For help to understand the arguments used above
```

Table S1. Primer sequences.

**Housekeeping marker**	L14	F: 5'-GGCTTTAGTGGATGGACCCT-3'<br>R: 5'-ATTGATATCCGCCTTCTCCC-3'
**Cardiac injury markers**	Nppb	F: 5'-GAG TCC TTC GGT CTC AAG GC-3'<br>R: 5'-ACT TCA GTG CGT TAC AGC CC-3'
	Fabp3	F: 5'-AGA GTT CGA CGA GGT GAC AG-3'<br>R: 5'-TTG TCT CCT GCC CGT TCC AC-3'
	Tnnt2	F: 5'-GCT ACA GAC TCT GAT CGA GGC T-3'<br>R: 5'-CTC ATT GCG AAT ACG CTG CTG-3'
	Anxa1	F: 5'-GTA TCC TCG GAT GTT GCT GCC-3'<br>R: 5'-CCA TTC TCC TGT AAG TAC GCG G-3'
	Acta2	F: 5'-GCTGGTGATGATGCTCCCA-3'<br>R: 5'-GCCCATTCCAACCATTACTCC-3'
	Col3a1	F: 5'-CCC TGG ACC TCA GGG TAT CA-3'<br>R: 5'-GGG TTT CCA TCC CTT CCA GG-3'
	Col1a1	F: 5'-CCAAGAAGACATCCCTGAAGTCA-3'<br>R: 5'-TGCACGTCATCGCACACA-3'
**RAAS-associated genes**	Ren	F: 5'-ACATGACCAGGCTCAGTGCTGA-3'<br>R: 5'-TACCGATGCCAATCTCGCCGTA-3'
	Agtr1	F: 5'-GCCATTGTCCACCCGATGAAGT-3'<br>R: 5'-ACACATTTCGGTGGATGACGGC-3'
	Agtr2	F: 5'-GCCACCAGCAGAAACATTACC-3'<br>R: 5'-ACAACAAAACAGTGAGACCACAAC-3'
	Scnn1a	F: 5'-TGATGGTGGCTTCAACGTGAGG-3'<br>R: 5'-AGTGCAGTCTCCGTAGTTGCCT-3'
	Slc9a3	F: 5'-TCTGTTTGTCAGCACCACTCTCA-3'<br>R: 5'-TCACGATGCTCGCTCCTCTTCA-3'
	Aqp1	F: 5'-CTTGCCATTGGCTTGTCTGTGG-3'<br>R: 5'-CCAGTGGTTTGAGAAGTTGCGG-3'
	Aqp2	F: 5'-GCCATCCTCCATGAGATTACCC-3'<br>R: 5'-CGCTCATCAGTGGAGGCAAAGA-3'
**Aldosterone-induced genes**	Sgk1	F: 5'-CTCATTCCAGACCGCTGACAAGC-3'<br>R: 5'-CCAGGGCACTGGCTATTTCAGC-3'
	Crip1	F: 5'-ATCGTCCCTGCCTGAAGTGTGA-3'<br>R: 5'-CCCAAACATGGCGGAGTAGCAG-3'
	Ank	F: 5'-CGTGGACTCATGCTGGCATTCT-3'<br>R: 5'-GTTCTCGGCATTCCAGGTGACT-3'
	Cited4	F: 5'-ACACCGAGCTCATCGACGAAGA-3'<br>R: 5'-GTCTGAGAAGCAATCGAACTCGC-3'

	Lcn2	F: 5'-TTT CAC CCG CTT TGC CAA GT-3'<br>R: 5'-GTC TCT GCG CAT CCC AGT CA-3'
**Inflammation-related genes**	Tnfa	F: 5'-CCC TCA CAC TCA CAA ACC ACC-3'<br>R: 5'-GCC TTG TCC CTT GAA GAG AAC C-3'
	Il6	F: 5'-TCT GAA GGA CTC TGG CTT TG-3'<br>R: 5'-GAT GGA TGC TAC CAA ACT GGA-3'
	Infg	F: 5'-ACT CAA GTG GCA TAG ATG TGG AAG-3'<br>R: 5'-GAC GCT TAT GTT GTT GCT GAT GG-3'
	Cd86	F: 5'-AGA ACT TAC GGA AGC ACC CAC-3'<br>R: 5'-CTG CCA AAA TAC TAC CAG CTC AC-3'
	Tgfb1	F: 5'-CTC CCG TGG CTT CTA GTG C-3'<br>R: 5'-GCC TTA GTT TGG ACA GGA TCT G-3'
**Target genes in mesenteric artery**	Adra1a	F: 5'-TGGCTGCCATTCTTCCTCGTGA-3'<br>R: 5'-TTCTTGAACTCCTGGCTGGAGC-3'
	Chrm1	F: 5'-CAGAAGTGGTGATCAAGATGCCTAT -3'<br>R: 5'-GAGCTTTTGGGAGGCTGCTT-3'
	Chrm3	F: 5'-CAGGCAGTTCTCGAAGCTGTAG-3'<br>R: 5'-GTCTTGGTCCATCTGCTCAGCA-3'
	Vwf	F: 5'-CTTCTGTACGCCTCAGCTATG-3'<br>R: 5'-GCCGTTGTAATTCCCACACAAG-3'
	Egr1	F: 5'-AGCGAACAACCCTATGAGCACC-3'<br>R: 5'-ATGGGAGGCAACCGAGTCGTTT-3'
	Klf4	Qiagene
	Sphk1	F: 5'-GGTGAATGGGCTAATGGAACG-3'<br>R: 5'-CTGCTCGTACCCAGCATAGTG-3'
	Nos3	F: 5'-CGCAAGAGGAAGGAGTCTAGCA<br>R: 5'-TCGAGCAAAGGCACAGAAGTGG

Table S2. Statistics.

Figure	Parameter	ND M	N	HSD M	N	ND F	N	HSD F	N	Shapiro-Wilk Test (Normality)	Comparison Test	Statistical Outcome (ANOVA statistic)	Statistical Outcome (post hoc)
1b	Systolic Blood Pressure	117.80 ± 3.08	6	##### ± 3.00	5	122.10 ± 3.07	7	132.60 ± 2.71	7	$W_{ND,M}(6)$= 0.88, p = 0.28; $W_{HSD,M}(5)$= 0.87, p = 0.27; $W_{ND,F}(7)$= 0.93, p = 0.52; $W_{HSD,F}(7)$= 0.94, p = 0.62	Two-way ANOVA + Fisher LSD	Int: F(1,21) = 2.71, p = 0.115; Sex: F(1,21) = 9.33, p = 0.006; Diet: F(1,21) = 3.48, p = 0.076	ND,M vs. HSD,M p=0.885; ND,F vs. HSD,F p=0.015
1c	Heart rate (bpm)	577.40 ± ####	5	##### ± 16.91	6	557.50 ± 16.84	6	543.00 ± 16.00	5	$W_{ND,M}(5)$= 0.97, p = 0.90; $W_{HSD,M}(6)$= 0.97; p = 0.89, $W_{ND,F}(6)$= 0.95, p = 0.73; $W_{HSD,F}(5)$= 0.85, p = 0.19	Two-way ANOVA + Fisher LSD	Int: F(1,18) = 1.36, p = 0.258; Sex: F(1,18) = 0.001, p = 0.0001; Diet: F(1,18) = 4.17, p = 0.056	ND,M vs. HSD,M p=0.036; ND,F vs. HSD,F p=0.544
1d	Heart-to-body weight ratio	5.71 ± 0.43	6	6.23 ± 0.28	5	4.16 ± 0.19	7	5.79 ± 0.17	10	$W_{ND,M}(6)$= 0.95, p = 0.74; $W_{HSD,M}(5)$= 0.85, p = 0.21; $W_{ND,F}(7)$= 0.96, p = 0.86; $W_{HSD,F}(7)$= 0.96, p = 0.79	Two-way ANOVA + Fisher LSD	Int: F(1,24) = 4.31, p = 0.049; Sex: F(1,24) = 13.79, p = 0.0011; Diet: F(1,24) = 16.13, p = 0.0005	ND,M vs. HSD,M p=0.223; ND,F vs. HSD,F p<0.0001
2b	Stroke Volume	27.00 ± 8.50	5	35.00 ± 7.00	5	20.00 ± 10.00	5	23.00 ± 6.00	5	$W_{ND,M}(5)$= 0.86, p = 0.23; $W_{HSD,M}(5)$= 0.95, p = 0.75; $W_{ND,F}(5)$= 0.64, p = 0.0022; $W_{HSD,F}(5)$= 0.89, p = 0.38	Two-way ANOVA + Fisher LSD	Int: F(1,16) = 2.94, p = 0.106; Sex: F(1,16) = 7.26, p = 0.016; Diet: F(1,16) = 3.84, p = 0.068	ND,M vs. HSD,M p=0.019; ND,F vs. HSD,F p=0.865
2c	End Diastolic Volume	41.40 ± 3.56	5	52.80 ± 3.89	5	34.00 ± 3.54	5	34.80 ± 2.27	5	$W_{ND,M}(5)$= 0.85, p = 0.20; $W_{HSD,M}(5)$= 0.92, p = 0.52; $W_{ND,F}(5)$= 0.88, p = 0.29; $W_{HSD,F}(5)$= 0.91, p = 0.46	Two-way ANOVA + Fisher LSD	Int: F(1,16) = 2.47, p = 0.135; Sex: F(1,16) = 14.20, p = 0.0017; Diet: F(1,16) = 3.28, p = 0.089	ND,M vs. HSD,M p=0.029; ND,F vs. HSD,F p=0.869
2d	End Systolic Volume	15.00 ± 1.14	5	18.00 ± 2.61	5	11.00 ± 1.58	5	9.60 ± 1.33	5	$W_{ND,M}(5)$= 0.85, p = 0.21; $W_{HSD,M}(5)$= 0.89, p = 0.35; $W_{ND,F}(5)$= 0.84, p = 0.15; $W_{HSD,F}(5)$= 0.90, p = 0.39	Two-way ANOVA + Fisher LSD	Int: F(1,16) = 1.57, p = 0.229; Sex: F(1,16) = 12.44, p = 0.0028; Diet: F(1,16) = 0.27, p = 0.655	ND,M vs. HSD,M p=0.245; ND,F vs. HSD,F p=0.581
2f	End Diastolic LV mass	103.00 ± ####	5	##### ± 38.50	5	83.00 ± 19.50	5	85.00 ± 29.00	5	$W_{ND,M}(5)$= 0.77, p = 0.042; $W_{HSD,M}(5)$= 0.999, p = 0.999; $W_{ND,F}(5)$= 0.96, p = 0.79; $W_{HSD,F}(5)$= 0.998, p = 0.99	Two-way ANOVA + Fisher LSD	Int: F(1,16) = 2.30, p = 0.149; Sex: F(1,16) = 13.91, p = 0.002; Diet: F(1,16) = 3.56, p = 0.078	ND,M vs. HSD,M p=0.029; ND,F vs. HSD,F p=0.798
2g	Relative LV wall mass	2.43 ± 0.11	5	2.38 ± 0.24	5	2.52 ± 0.20	5	2.44 ± 0.10	5	$W_{ND,M}(5)$= 0.96, p = 0.84; $W_{HSD,M}(5)$= 0.94, p = 0.67; $W_{ND,F}(5)$= 0.89, p = 0.35; $W_{HSD,F}(5)$= 0.85, p = 0.20	Two-way ANOVA + Fisher LSD	Int: F(1,16) = 0.02, p = 0.91; Sex: F(1,16) = 0.19, p = 0.67; Diet: F(1,16) = 0.16, p = 0.70	ND,M vs. HSD,M p=0.849; ND,F vs. HSD,F p=0.721
2h	% Ejection fraction	63.80 ± 1.99	5	66.20 ± 3.31	5	66.60 ± 4.31	5	72.20 ± 3.25	5	$W_{ND,M}(5)$= 0.87, p = 0.26; $W_{HSD,M}(5)$= 0.79, p = 0.06; $W_{ND,F}(5)$= 0.98, p = 0.91; $W_{HSD,F}(5)$= 0.92, p = 0.50	Two-way ANOVA + Fisher LSD	Int: F(1,16) = 0.23, p = 0.64; Sex: F(1,16) = 1.76, p = 0.20; Diet: F(1,16) = 1.46, p = 0.25	ND,M vs. HSD,M p=0.616; ND,F vs. HSD,F p=0.250
3b	Aorta wall thickness	65.97 ± ####	6	93.25 ± 23.75	5	79.42 ± 22.05	8	81.66 ± 27.08	9	$W_{ND,M}(6)$= 0.79, p = 0.052; $W_{HSD,M}(5)$= 0.91, p = 0.44; $W_{ND,F}(8)$= 0.94, p = 0.61; $W_{HSD,F}(9)$= 0.80, p = 0.02	Two-way ANOVA + Fisher LSD	Int: F(1,24) = 3.32, p = 0.081; Sex: F(1,24) = 0.75, p = 0.396; Diet: F(1,24) = 6.87, p = 0.015	ND,M vs. HSD,M p=0.009; ND,F vs. HSD,F p=0.529
3d	Collagen deposition aorta (Von Gieson)	150.70 ± 9.27	6	##### ± 25.94	5	95.75 ± 7.59	5	222.70 ± 28.31	9	$W_{ND,M}(6)$= 0.88, p = 0.26; $W_{HSD,M}(5)$= 0.93, p = 0.61; $W_{ND,F}(5)$= 0.88, p = 0.32; $W_{HSD,F}(9)$= 0.94, p = 0.63	Two-way ANOVA + Fisher LSD	Int: F(1,23) = 7.72, p = 0.011; Sex: F(1,23) = 0.30, p = 0.591; Diet: F(1,23) = 5.70, p = 0.027	ND,M vs. HSD,M p=0.793; ND,F vs. HSD,F p=0.001
3f	Smooth muscle actin - aorta	62.84 ± 4.95	6	48.90 ± 2.76	5	64.45 ± 1.91	7	64.22 ± 2.03	9	$W_{ND,M}(6)$= 0.93, p = 0.55; $W_{HSD,M}(5)$= 0.94, p = 0.65; $W_{ND,F}(7)$= 0.95, p = 0.69; $W_{HSD,F}(9)$= 0.93, p = 0.44	Two-way ANOVA + Fisher LSD	Int: F(1,23) = 5.22, p = 0.032; Sex: F(1,23) = 7.94, p = 0.010; Diet: F(1,23) = 5.56, p = 0.027	ND,M vs. HSD,M p=0.006; ND,F vs. HSD,F p=0.954
3g	Osteopontin - aorta	5.78 ± 0.33	6	8.99 ± 0.59	5	7.44 ± 0.44	##	6.67 ± 0.25	8	$W_{ND,M}(5)$= 0.92, p = 0.53; $W_{HSD,M}(5)$= 0.84, p = 0.17; $W_{ND,F}(5)$= 0.93, p = 0.40; $W_{HSD,F}(5)$= 0.96, p = 0.76	Two-way ANOVA + Fisher LSD	Int: F(1,25) = 21.31, p = 0.0001; Sex: F(1,25) = 0.58, p = 0.454; Diet: F(1,25) = 8.03, p = 0.009	ND,M vs. HSD,M p<0.0001; ND,F vs. HSD,F p=0.161
4a	Max constriction (KCl)	5.97 ± 0.22	5	4.25 ± 0.59	5	4.65 ± 0.43	7	4.42 ± 0.42	9	$W_{ND,M}(5)$= 0.92, p = 0.54; $W_{HSD,M}(5)$= 0.80, p = 0.09; $W_{ND,F}(7)$= 0.88, p = 0.22; $W_{HSD,F}(9)$= 0.93, p = 0.44	Two-way ANOVA + Fisher LSD	Int: F(1,22) = 2.64, p = 0.119; Sex: F(1,22) = 1.53, p = 0.229; Diet: F(1,22) = 4.49, p = 0.046	ND,M vs. HSD,M p=0.026; ND,F vs. HSD,F p=0.695
4b - M	Contraction to cirazolin (10^9)	-0.16 ± 0.06	5	-0.01 ± 0.05	5					$W_{ND,M}(12)$= 0.87, p = 0.07; $W_{HSD,M}(10)$= 0.89, p = 0.16	RM two-way ANOVA + Fisher LSD	Int: F(6,120) = 6.63, p < 0.0001; Cirazolin concentration: F(3.090,61.81) = 181.4, p < 0.0001; Diet: F(1,20) = 5.978, p = 0.024; Subject: F(20,120) = 12.30, p < 0.0001	ND,M vs. HSD,M p=0.081
	Contraction to cirazolin (10^8)	0.01 ± 1.03	5	0.13 ± 2.21	5					$W_{ND,M}(12)$= 0.66, p = 0.0003; $W_{HSD,M}(10)$= 0.79, p = 0.01	"	"	ND,M vs. HSD,M p=0.890
	Contraction to cirazolin (3*10^8)	4.93 ± 0.32	5	3.61 ± 0.68	5					$W_{ND,M}(12)$= 0.94, p = 0.52; $W_{HSD,M}(10)$= 0.93, p = 0.49	"	"	ND,M vs. HSD,M p=0.105
	Contraction to cirazolin (10^7)	6.45 ± 1.15	5	5.67 ± 2.62	5					$W_{ND,M}(12)$= 0.96, p = 0.83; $W_{HSD,M}(10)$= 0.84, p = 0.04	"	"	ND,M vs. HSD,M p=0.030
	Contraction to cirazolin (3*10^7)	6.55 ± 0.29	5	4.79 ± 0.49	5					$W_{ND,M}(12)$= 0.96, p = 0.71; $W_{HSD,M}(10)$= 0.86, p = 0.07	"	"	ND,M vs. HSD,M p=0.007
	Contraction to cirazolin (10^6)	6.40 ± 0.29	5	4.14 ± 0.58	5					$W_{ND,M}(12)$= 0.93, p = 0.40; $W_{HSD,M}(10)$= 0.91, p = 0.27	"	"	ND,M vs. HSD,M p=0.004
	Contraction to cirazolin (3*10^6)	6.18 ± 0.31	5	4.56 ± 0.51	5					$W_{ND,M}(12)$= 0.90, p = 0.16; $W_{HSD,M}(10)$= 0.86, p = 0.08	"	"	ND,M vs. HSD,M p=0.017
4b - F	Contraction to cirazolin (10^9)					-0.11 ± 0.06	7	-0.14 ± 0.06	9	$W_{ND,F}(14)$= 0.92, p = 0.246; $W_{HSD,F}(19)$= 0.95, p = 0.353	RM two-way ANOVA + Fisher LSD	Int: F(6,186) = 1.18, p = 0.318; Cirazolin concentration: F(2.541,78.76) = 183.5, p < 0.0001; Diet: F(1,31) = 0.820, p = 0.372; Subject: F(31,186) = 9.74, p < 0.0001	ND,F vs. HSD,F p=0.765
	Contraction to cirazolin (10^8)					1.81 ± 4.90	7	1.55 ± 5.55	9	$W_{ND,F}(14)$= 0.87, p = 0.042; $W_{HSD,F}(19)$= 0.82, p = 0.002	"	"	ND,F vs. HSD,F p=0.959
	Contraction to cirazolin (3*10^8)					5.04 ± 0.32	7	5.19 ± 0.47	9	$W_{ND,F}(14)$= 0.90, p = 0.107; $W_{HSD,F}(19)$= 0.94, p = 0.225	"	"	ND,F vs. HSD,F p=0.789
	Contraction to cirazolin (10^7)					5.91 ± 0.35	7	6.05 ± 0.44	9	$W_{ND,F}(14)$= 0.90, p = 0.126; $W_{HSD,F}(19)$= 0.97, p = 0.685	"	"	ND,F vs. HSD,F p=0.802
	Contraction to cirazolin (3*10^7)					6.02 ± 0.39	7	6.81 ± 0.36	9	$W_{ND,F}(14)$= 0.89, p = 0.095; $W_{HSD,F}(19)$= 0.98, p = 0.923	"	"	ND,F vs. HSD,F p=0.145
	Contraction to cirazolin (10^6)					5.96 ± 0.31	7	6.74 ± 0.30	9	$W_{ND,F}(14)$= 0.90, p = 0.116; $W_{HSD,F}(19)$= 0.94, p = 0.252	"	"	ND,F vs. HSD,F p=0.082
	Contraction to cirazolin (3*10^6)					5.72 ± 0.31	7	6.63 ± 0.26	9	$W_{ND,F}(14)$= 0.8917, p = 0.147; $W_{HSD,F}(19)$= 0.98, p = 0.877	"	"	ND,F vs. HSD,F p=0.034
4c - M	Dilation to carbachol (10^9) (KCl)	110.10 ± 4.60	5	93.25 ± 35.55	5					$W_{ND,M}(12)$= 0.75, p = 0.0025; $W_{HSD,M}(10)$= 0.92, p = 0.343	RM two-way ANOVA + Fisher LSD	Int: F(4,80) = 2.45, p = 0.053; Carbachol concentration: F(1.774,35.48) = 80.18, p < 0.0001; Diet: F(1,20) = 4.70, p = 0.043; Subject: F(20,80) = 21.18, p < 0.0001	ND,M vs. HSD,M p=0.028
	Dilation to carbachol (10^8) (KCl)	111.70 ± 7.10	5	87.97 ± 43.28	5					$W_{ND,M}(12)$= 0.71, p = 0.0012; $W_{HSD,M}(10)$= 0.90, p = 0.243		"	ND,M vs. HSD,M p=0.020
	Dilation to carbachol (10^7) (KCl)	111.70 ± 7.50	5	82.40 ± 49.61	5					$W_{ND,M}(12)$= 0.69, p = 0.0008; $W_{HSD,M}(10)$= 0.91, p = 0.304		"	ND,M vs. HSD,M p=0.033
	Dilation to carbachol (10^6) (KCl)	106.20 ± 9.30	5	76.51 ± 60.44	5					$W_{ND,M}(12)$= 0.73, p = 0.0015; $W_{HSD,M}(10)$= 0.91, p = 0.307		"	ND,M vs. HSD,M p=0.037
	Dilation to carbachol (10^5) (KCl)	54.99 ± 7.65	5	44.65 ± 9.83	5					$W_{ND,M}(12)$= 0.96, p = 0.846; $W_{HSD,M}(10)$= 0.89, p = 0.174		"	ND,M vs. HSD,M p=0.418
4c - F	Dilation to carbachol (10^9) (KCl)					107.50 ± 1.45	7	106.10 ± 2.14	9	$W_{ND,F}(16)$= 0.93, p = 0.224; $W_{HSD,F}(20)$= 0.96, p = 0.609	RM two-way ANOVA + Fisher LSD	Int: F(4,136) = 0.95, p = 0.438; Carbachol concentration: F(1.373,46.69) = 184.5, p < 0.0001; Diet: F(1,34) = 0.81, p = 0.375; Subject: F(34,136) = 6.23, p < 0.0001	ND,F vs. HSD,F p=0.589
	Dilation to carbachol (10^8) (KCl)					110.80 ± 1.69	7	108.50 ± 2.47	9	$W_{ND,F}(16)$= 0.94, p = 0.402; $W_{HSD,F}(20)$= 0.96, p = 0.563		"	ND,F vs. HSD,F p=0.458
	Dilation to carbachol (10^7) (KCl)					110.50 ± 2.03	7	108.90 ± 2.65	9	$W_{ND,F}(16)$= 0.95, p = 0.498; $W_{HSD,F}(20)$= 0.92, p = 0.097		"	ND,F vs. HSD,F p=0.633
	Dilation to carbachol (10^6) (KCl)					106.40 ± 21.53	7	107.70 ± 41.52	9	$W_{ND,F}(16)$= 0.88, p = 0.044; $W_{HSD,F}(20)$= 0.86, p = 0.009		"	ND,F vs. HSD,F p=0.584
	Dilation to carbachol (10^5) (KCl)					62.26 ± 27.41	7	49.45 ± 28.33	9	$W_{ND,F}(16)$= 0.84, p = 0.011; $W_{HSD,F}(20)$= 0.95, p = 0.300		"	ND,F vs. HSD,F p=0.247
4d	Plasma nitrite concentration	20.95 ± 2.91	6	8.18 ± 1.04	5	19.20 ± 3.39	##	16.85 ± 2.18	##	$W_{ND,M}(6)$= 0.85, p = 0.15; $W_{HSD,M}(5)$= 0.85, p = 0.18; $W_{ND,F}(7)$= 0.96, p = 0.84; $W_{HSD,F}(10)$= 0.88, p = 0.16	Two-way ANOVA + Fisher LSD	Int: F(1,24) = 3.60, p = 0.070; Sex: F(1,24) = 1.589, p = 0.220; Diet: F(1,24) = 7.59, p = 0.011	ND,M vs. HSD,M p=0.006; ND,F vs. HSD,F p=0.504
5c	Bowman capsule area (μm^2)	5000 ± 219	5	6237 ± 279	5	4904 ± 276	7	5160 ± 270	9	$W_{ND,M}(5)$= 0.84, p = 0.153; $W_{HSD,M}(5)$= 0.95, p = 0.710; $W_{ND,F}(7)$= 0.95, p = 0.691; $W_{HSD,F}(9)$= 0.98, p = 0.946	Two-way ANOVA + Fisher LSD	Int: F(1,22) = 2.96, p = 0.100; Sex: F(1,22) = 4.22, p = 0.052; Diet: F(1,22) = 6.85, p = 0.016	ND,M vs. HSD,M p=0.011; ND,F vs. HSD,F p=0.479
5d	Vessel wall thickness (10-40µm)	4.46 ± 0.37	5	4.66 ± 0.25	5	3.93 ± 0.30	7	5.37 ± 0.25	9	$W_{ND,M}(5)$= 0.94, p = 0.677; $W_{HSD,M}(5)$= 0.96, p = 0.794; $W_{ND,F}(7)$= 0.95, p = 0.759; $W_{HSD,F}(9)$= 0.96, p = 0.749	Two-way ANOVA + Fisher LSD	Int: F(1,22) = 4.29, p = 0.0502; Sex: F(1,22) = 0.09, p = 0.765; Diet: F(1,22) = 7.39, p = 0.013	ND,M vs. HSD,M p=0.683; ND,F vs. HSD,F p=0.001
5e	Vessel wall thickness (40-80µm)	6.11 ± 0.45	5	6.76 ± 0.44	5	5.94 ± 0.50	7	7.84 ± 0.38	9	$W_{ND,M}(5)$= 0.94, p = 0.651; $W_{HSD,M}(5)$= 0.89, p = 0.349; $W_{ND,F}(7)$= 0.95, p = 0.739; $W_{HSD,F}(9)$= 0.84, p = 0.062	Two-way ANOVA + Fisher LSD	Int: F(1,22) = 1.83, p = 0.190; Sex: F(1,22) = 0.96, p = 0.337; Diet: F(1,22) = 7.60, p = 0.012	ND,M vs. HSD,M p=0.379; ND,F vs. HSD,F p=0.003
S1a	Body weight (baseline - month 0)	31.83 ± 0.74	7	31.95 ± 1.05	6					$W_{ND,M}(7)$= 0.92, p = 0.507; $W_{HSD,M}(6)$= 0.87, p = 0.230	Two-way ANOVA + Fisher LSD	Time: F(1.909,19.41) = 2.49, p = 0.111; Diet: F(1,11) = 18.49, p = 0.001; Time x Diet: F(6,61) = 3.04, p = 0.012	ND,M vs. HSD,M p=0.927
	Body weight (month 1)	31.94 ± 0.60	7	29.88 ± 0.91	6					$W_{ND,M}(7)$= 0.93, p = 0.554; $W_{HSD,M}(6)$= 0.93, p = 0.611	"	"	ND,M vs. HSD,M p=0.092
	Body weight (month 3)	32.77 ± 0.69	7	29.77 ± 0.94	6					$W_{ND,M}(7)$= 0.84, p = 0.105; $W_{HSD,M}(6)$= 0.90, p = 0.355	"	"	ND,M vs. HSD,M p=0.028
	Body weight (month 7)	32.41 ± 0.40	7	27.90 ± 0.76	5					$W_{ND,M}(7)$= 0.89, p = 0.285; $W_{HSD,M}(5)$= 0.788, p = 0.064	"	"	ND,M vs. HSD,M p=0.002
	Body weight (month 9)	33.47 ± 0.60	7	29.06 ± 0.55	5					$W_{ND,M}(7)$= 0.95, p = 0.774; $W_{HSD,M}(5)$= 0.84, p = 0.169	"	"	ND,M vs. HSD,M p=0.0003
	Body weight (month 12)	34.31 ± 0.73	7	29.68 ± 0.86	5					$W_{ND,M}(7)$= 0.97, p = 0.892; $W_{HSD,M}(5)$= 0.787, p = 0.064	"	"	ND,M vs. HSD,M p=0.003
	Body weight (month 14)	33.80 ± 0.91	6	30.98 ± 1.16	5					$W_{ND,M}(6)$= 0.93, p = 0.616; $W_{HSD,M}(5)$= 0.81, p = 0.099	"	"	ND,M vs. HSD,M p=0.091
S1b	Body weight (baseline - month 0)					24.89 ± 0.57	9	24.51 ± 0.28	##	$W_{ND,F}(9)$= 0.98, p = 0.952; $W_{HSD,F}(10)$= 0.94, p = 0.558	Two-way ANOVA + Fisher LSD	Time: F(2.315,38.58) = 44.80, p < 0.0001; Diet: F(1,17) = 22.15, p = 0.0002; Time x Diet: F(6,100) = 15.14, p < 0.0001	ND,F vs. HSD,F p=0.562
	Body weight (month 1)					25.11 ± 0.73	9	23.45 ± 0.44	##	$W_{ND,F}(9)$= 0.90, p = 0.264; $W_{HSD,F}(10)$= 0.91, p = 0.258	"	"	ND,F vs. HSD,F p=0.072
	Body weight (month 3)					26.43 ± 0.74	9	24.46 ± 0.44	##	$W_{ND,F}(9)$= 0.90, p = 0.276; $W_{HSD,F}(10)$= 0.94, p = 0.529	"	"	ND,F vs. HSD,F p=0.039
	Body weight (month 7)					29.41 ± 1.15	9	24.36 ± 0.47	##	$W_{ND,F}(9)$= 0.94, p = 0.550; $W_{HSD,F}(10)$= 0.92, p = 0.327	"	"	ND,F vs. HSD,F p=0.002
	Body weight (month 9)					31.62 ± 1.40	9	25.47 ± 0.55	##	$W_{ND,F}(9)$= 0.93, p = 0.516; $W_{HSD,F}(10)$= 0.95, p = 0.698	"	"	ND,F vs. HSD,F p=0.002
	Body weight (month 12)					32.70 ± 1.32	9	26.48 ± 0.61	##	$W_{ND,F}(9)$= 0.92, p = 0.369; $W_{HSD,F}(10)$= 0.94, p = 0.523	"	"	ND,F vs. HSD,F p=0.001
	Body weight (month 14)					34.71 ± 1.37	7	26.51 ± 0.55	##	$W_{ND,F}(7)$= 0.97, p = 0.864; $W_{HSD,F}(10)$= 0.96, p = 0.815	"	"	ND,F vs. HSD,F p=0.001
S1c	Food intake/week (g)	26.51 ± 0.59	4	37.00 ± 2.80	4	21.04 ± 0.76	4	26.28 ± 1.47	4	$W_{ND,M}(4)$= 0.91, p = 0.478; $W_{HSD,M}(4)$= 0.88, p = 0.345; $W_{ND,F}(4)$= 0.97, p = 0.839; $W_{HSD,F}(4)$= 0.99, p = 0.957	Two-way ANOVA + Fisher LSD	Int: F(1,21) = 2.53, p = 0.138; Sex: F(1,12) = 24.00, p = 0.0004; Diet: F(1,12) = 22.64, p = 0.0005	ND,M vs. HSD,M p=0.001; ND,F vs. HSD,F p=0.045
S1d	Water intake/week (g)	29.34 ± 1.50	4	##### ± 4.93	4	29.78 ± 1.74	4	95.99 ± 4.65	4	$W_{ND,M}(4)$= 0.89, p = 0.371; $W_{HSD,M}(4)$= 0.96; p = 0.803, $W_{ND,F}(4)$= 0.95, p = 0.686; $W_{HSD,F}(4)$= 0.99, p = 0.976	Two-way ANOVA + Fisher LSD	Int: F(1,12) = 0.41, p = 0.535; Sex: F(1,12) = 0.26, p = 0.616; Diet: F(1,12) = 366.1, p < 0.0001	ND,M vs. HSD,M p < 0.0001; ND,F vs. HSD,F p < 0.0001

S3a	#nuclei/SMA+ area	0.22 ± 0.00	6	0.18 ± 0.01	5	0.23 ± 0.01	6	0.21 ± 0.01	9	$W_{ND,M}$(6)= 0.96, p = 0.845; $W_{HSD,M}$(5)= 0.94, p = 0.656; $W_{ND,F}$(6)= 0.93, p = 0.579; $W_{HSD,F}$(10)= 0.92, p = 0.33	Two-way ANOVA + Fisher LSD	Int: F(1,23) = 0.80, p = 0.382; Sex: F(1,23) = 4.46, **p = 0.046**; Diet: F(1,23) = 10.95, **p = 0.003**	ND,M vs. HSD,M **p=0.011**; ND,F vs. HSD,F p=0.075
S3b	SMA:OPN ratio	**4.30 ± 0.40**	**6**	**2.20 ± 0.87**	**5**	**1.93 ± 0.43**	**##**	**2.15 ± 0.30**	**8**	$W_{ND,M}$(6)= 0.93, p = 0.597; $W_{HSD,M}$(5)= 0.93, p = 0.610; $W_{ND,F}$(10)= 0.79, **p = 0.011;** $W_{HSD,F}$(8)= 0.93, p = 0.52	Two-way ANOVA + Fisher LSD	Int: F(1,25) = 50.53, **p < 0.0001**; Sex: F(1,25) = 63.30, **p < 0.0001**; Diet: F(1,25) = 38.50, **p < 0.0001**	ND,M vs. HSD,M **p<0.0001**; ND,F vs. HSD,F p=0.471
S3c	CD68 positivity	8.65 ± 0.26	6	8.17 ± 0.56	5	10.07 ± 0.69	6	9.18 ± 0.57	9	$W_{ND,M}$(6)= 0.88, p = 0.275; $W_{HSD,M}$(5)= 0.81, p = 0.089; $W_{ND,F}$(6)= 0.91, p = 0.414; $W_{HSD,F}$(9)= 0.94, p = 0.558	Two-way ANOVA + Fisher LSD	Int: F(1,22) = 0.13, p = 0.725; Sex: F(1,22) = 4.36, **p = 0.049**; Diet: F(1,22) = 1.41, p = 0.248	ND,M vs. HSD,M p=0.587; ND,F vs. HSD,F p=0.252
S4a	Max constriction (Cirazolin)	6.25 ± 0.51	5	4.46 ± 0.49	5	5.95 ± 0.66	7	6.55 ± 0.38	9	$W_{ND,M}$(5)= 0.98, p = 0.940; $W_{HSD,M}$(5)= 0.94, p = 0.652; $W_{ND,F}$(7)= 0.859, p = 0.148; $W_{HSD,F}$(9)= 0.97, p = 0.89	Two-way ANOVA + Fisher LSD	Int: F(1,22) = 4.92, **p = 0.037**; Sex: F(1,22) = 2.76, p = 0.111; Diet: F(1,22) = 1.25, p = 0.277	ND,M vs. HSD,M **p=0.045**; ND,F vs. HSD,F p=0.386
S4b	Contraction to cirazolin (10^9)			-0.01 ± 0.05	5			-0.14 ± 0.06	9	$W_{HSD,M}$(10)= 0.89, p = 0.16; $W_{HSD,F}$(19)= 0.95, p = 0.35	RM two-way ANOVA + Fisher LSD	Int: F(6,162) = 4.88, **p = 0.0001**; Cirazolin concentration: F(2.841,76.72) = 137.7, **p < 0.0001**; Sex: F(1,27) = 7.747, **p = 0.010**; Subject: F(27,162) = 13.20, **p < 0.0001**	HSD,F vs. HSD,M p=0.101
	Contraction to cirazolin (10^8)			**0.13 ± 2.21**	**5**			**1.55 ± 5.55**	**9**	$W_{HSD,M}$(10)= 0.79, **p = 0.01**; $W_{HSD,F}$(19)= 0.82, **p = 0.002**	"	"	HSD,F vs. HSD,M p=0.082
	Contraction to cirazolin (3*10^8)			3.61 ± 0.68	5			5.19 ± 0.47	9	$W_{HSD,M}$(10)= 0.93, p = 0.49; $W_{HSD,F}$(19)= 0.94, p = 0.23	"	"	HSD,F vs. HSD,M p=0.073
	Contraction to cirazolin (10^7)			**5.67 ± 2.62**	**5**			**6.10 ± 2.37**	**9**	$W_{HSD,M}$(10)= 0.84, **p = 0.04**; $W_{HSD,F}$(19)= 0.97, p = 0.68	"	"	HSD,F vs. HSD,M p=0.133
	Contraction to cirazolin (3*10^7)			4.79 ± 0.49	5			6.81 ± 0.36	9	$W_{HSD,M}$(10)= 0.86, p = 0.07; $W_{HSD,F}$(19)= 0.98, p = 0.92	"	"	HSD,F vs. HSD,M **p=0.003**
	Contraction to cirazolin (10^6)			4.14 ± 0.58	5			6.74 ± 0.30	9	$W_{HSD,M}$(10)= 0.91, p = 0.27; $W_{HSD,F}$(19)= 0.94, p = 0.25	"	"	HSD,F vs. HSD,M **p=0.0014**
	Contraction to cirazolin (3*10^6)			4.56 ± 0.51	5			6.63 ± 0.26	9	$W_{HSD,M}$(10)= 0.86, p = 0.08; $W_{HSD,F}$(19)= 0.98, p = 0.88	"	"	HSD,F vs. HSD,M **p=0.003**
S4c	Dilation to carbachol (10^9)			91.52 ± 5.55	5			106.10 ± 2.14	9	$W_{HSD,M}$(10)= 0.92, p = 0.343; $W_{HSD,F}$(20)= 0.96, p = 0.609	RM two-way ANOVA + Fisher LSD	Int: F(4,112) = 8.04, **p < 0.0001**; Carbachol concentration: F(1.657,46.40) = 110.3, **p < 0.0001**; Sex: F(1,28) = 7.643, **p = 0.010**; Subject: F(28,112) = 13.29, **p < 0.0001**	HSD,F vs. HSD,M **p=0.031**
	Dilation to carbachol (10^8)			85.22 ± 6.95	5			108.50 ± 2.47	9	$W_{HSD,M}$(10)= 0.90, p = 0.243; $W_{HSD,F}$(20)= 0.96, p = 0.563	"	"	HSD,F vs. HSD,M **p=0.009**
	Dilation to carbachol (10^7)			78.63 ± 8.71	5			108.90 ± 2.65	9	$W_{HSD,M}$(10)= 0.91, p = 0.304; $W_{HSD,F}$(20)= 0.92, p = 0.097	"	"	HSD,F vs. HSD,M **p=0.007**
	Dilation to carbachol (10^6)			**76.51 ± 60.44**	**5**			**107.70 ± 41.52**	**9**	$W_{HSD,M}$(10)= 0.91; p = 0.307; $W_{HSD,F}$(20)= 0.86, **p = 0.009**	"	"	HSD,F vs. HSD,M **p=0.023**
	Dilation to carbachol (10^5)			44.65 ± 9.83	5			45.07 ± 5.75	9	$W_{HSD,M}$(10)= 0.89; p = 0.174; $W_{HSD,F}$(20)= 0.95, p = 0.300	"	"	HSD,F vs. HSD,M p=0.971
S4d	Dilation to carbachol (10^9)			**##### ± 46.78**	**5**			**96.71 ± 26.55**	**9**	$W_{HSD,M}$(12)= 0.84, **p = 0.025**; $W_{HSD,F}$(18)= 0.94, p = 0.256	RM two-way ANOVA + Fisher LSD	Int: F(4,112) = 0.25, p = 0.910; Carbachol concentration: F(3.318,92.92) = 89.99, **p < 0.0001**; Sex: F(1,28) = 0.135, p = 0.716; Subject: F(28,112) = 7.30, **p < 0.0001**	HSD,F vs. HSD,M p=0.555
	Dilation to carbachol (10^8)			85.66 ± 7.38	5			87.84 ± 4.72	9	$W_{HSD,M}$(12)= 0.94, p = 0.528; $W_{HSD,F}$(18)= 0.95, p = 0.394	"	"	HSD,F vs. HSD,M p=0.805
	Dilation to carbachol (10^7)			77.00 ± 8.86	5			78.53 ± 6.25	9	$W_{HSD,M}$(12)= 0.96, p = 0.796; $W_{HSD,F}$(18)= 0.94, p = 0.290	"	"	HSD,F vs. HSD,M p=0.889
	Dilation to carbachol (10^6)			66.99 ± 9.31	5			73.26 ± 7.89	9	$W_{HSD,M}$(12)= 0.97, p = 0.908; $W_{HSD,F}$(18)= 0.94, p = 0.321	"	"	HSD,F vs. HSD,M p=0.612
	Dilation to carbachol (10^5)			**8.43 ± 11.43**	**5**			**3.07 ± 17.05**	**9**	$W_{HSD,M}$(12)= 0.745, **p = 0.002**; $W_{HSD,F}$(18)= 0.74, **p = 0.002**	"	"	HSD,F vs. HSD,M p=0.843
S4e	Dilation to carbachol (10^9)					76.62 ± 6.16	7	94.87 ± 6.96	9	$W_{ND,F}$(12)= 0.92, p = 0.308; $W_{HSD,F}$(18)= 0.94, p = 0.256	RM two-way ANOVA + Fisher LSD	Int: F(4,112) = 0.92, p = 0.458; Carbachol concentration: F(3.534,98.96) = 96.12, **p < 0.0001**; Diet: F(1,28) = 4.31, **p = 0.047**; Subject: F(28,112) = 5.49, **p < 0.0001**	ND,F vs. HSD,F p=0.060
	Dilation to carbachol (10^8)					74.29 ± 6.09	7	87.84 ± 4.72	9	$W_{ND,F}$(12)= 0.87, p = 0.067; $W_{HSD,F}$(18)= 0.95, p = 0.394	"	"	ND,F vs. HSD,F p=0.092
	Dilation to carbachol (10^7)					71.20 ± 3.52	7	78.53 ± 6.25	9	$W_{ND,F}$(12)= 0.98, p = 0.993; $W_{HSD,F}$(18)= 0.94, p = 0.290	"	"	ND,F vs. HSD,F p=0.316
	Dilation to carbachol (10^6)					50.86 ± 7.27	7	73.26 ± 7.89	9	$W_{ND,F}$(12)= 0.92, p = 0.265; $W_{HSD,F}$(18)= 0.94, p = 0.321	"	"	ND,F vs. HSD,F **p=0.046**
	Dilation to carbachol (10^5)					**4.26 ± 5.61**	**7**	**3.07 ± 17.05**	**9**	$W_{ND,F}$(12)= 0.92, p = 0.321; $W_{HSD,F}$(18)= 0.74, **p = 0.002**	"	"	ND,F vs. HSD,F p=0.126
S4f	Dilation to carbachol (10^9)	**95.93 ± ####**	**5**	**##### ± 46.78**	**5**					$W_{ND,M}$(12)= 0.96, p = 0.825; $W_{HSD,M}$(12)= 0.84, **p = 0.025**	RM two-way ANOVA + Fisher LSD	Int: F(4,88) = 0.64, p = 0.638; Carbachol concentration: F(2.256,49.63) = 79.35, **p < 0.0001**; Diet: F(1,22) = 1.25, p = 0.275; Subject: F(22,88) = 6.92, **p < 0.0001**	ND,M vs. HSD,M p=0.308
	Dilation to carbachol (10^8)	89.30 ± 4.11	5	85.66 ± 7.38	5					$W_{ND,M}$(12)= 0.92, p = 0.274; $W_{HSD,M}$(12)= 0.94, p = 0.528	"	"	ND,M vs. HSD,M p=0.671
	Dilation to carbachol (10^7)	86.63 ± 3.32	5	77.00 ± 8.86	5					$W_{ND,M}$(12)= 0.92, p = 0.308; $W_{HSD,M}$(12)= 0.96, p = 0.796	"	"	ND,M vs. HSD,M p=0.326
	Dilation to carbachol (10^6)	**74.16 ± ####**	**5**	**67.03 ± 58.17**	**5**					$W_{ND,M}$(12)= 0.86, **p = 0.043**; $W_{HSD,M}$(12)= 0.97, p = 0.908	"	"	ND,M vs. HSD,M p=0.720
	Dilation to carbachol (10^5)	**17.39 ± ####**	**5**	**8.43 ± 11.43**	**5**					$W_{ND,M}$(12)= 0.81, **p = 0.025**; $W_{HSD,M}$(12)= 0.75, **p = 0.002**	"	"	ND,M vs. HSD,M p=0.126

Figure	Parameter	REV M base	N	REV M 6M	N	REV M 14M	N	REV F base	N	REV F 6M	N	REV F 14M	N	Shapiro-Wilk Test (Normality)	Comparison Test	Statistical Outcome (ANOVA statistic)	Statistical Outcome (post hoc)
6b	Systolic Blood Pressure	120.00 ± 3.28	6	123.40 ± 0.93	5	123.40 ± 0.93	5	122.30 ± 1.92	8	131.50 ± 1.96	8	119.20 ± 3.09	6	$W_{REV,M,base}$(6)= 0.83, p = 0.113; $W_{REV,M,6M}$(5)= 0.95, p = 0.754, $W_{REV,M,14M}$(5)= 0.95, p = 0.754, $W_{REV,F,base}$(7)= 0.85, p = 0.095, $W_{REV,F,6M}$(7)= 0.99, p = 0.995, $W_{REV,F,14M}$(7)= 0.94, p = 0.672	Mixed effects + Fisher LSD	Time: F(1.75,17.50) = 5.40, **p = 0.018**; Sex: F(1,12) = 0.9997, p = 0.337; Int: F(2,20) = 3.56, **p = 0.048**	M,base vs. M,6M p=0.369; M,base vs. M,14M p=0.598; M,6M vs. M,14M p=0.604; F,base vs. F,6M **p=0.015**; F,base vs. F,14M p=0.421; F,6M vs. F,14M **p=0.013**
6c	Stroke Volume	22.60 ± 2.98	5	33.20 ± 3.73	5	28.20 ± 2.85	5	20.83 ± 0.31	6	26.60 ± 1.94	5	25.60 ± 2.04	5	$W_{REV,M,base}$(5)= 0.87, p = 0.248; $W_{REV,M,6M}$(5)= 0.92, p = 0.524, $W_{REV,M,14M}$(5)= 0.82, p = 0.113, $W_{REV,F,base}$(6)= 0.87, p = 0.212, $W_{REV,F,6M}$(5)= 0.92, p = 0.556, $W_{REV,F,14M}$(5)= 0.96, p = 0.814	Mixed effects + Fisher LSD	Time: F(1.977,14.83) = 13.41, **p = 0.001**; Sex: F(1,10) = 1.87, p = 0.201; Int: F(2,15) = 1.13, p = 0.349	M,base vs. M,6M p=0.070; M,base vs. M,14M p=0.062; M,6M vs. M,14M p=0.513; F,base vs. F,6M **p=0.045**; F,base vs. F,14M p=0.078; F,6M vs. F,14M p=0.319
6d	End Diastolic LV mass	**93.50 ± 44.80**	**6**	**104.00 ± 33.00**	**5**	**83.00 ± 42.50**	**5**	**80.00 ± 3.00**	**6**	**86.00 ± 24.00**	**5**	**87.00 ± 14.00**	**5**	$W_{REV,M,base}$(6)= 0.96, p = 0.856; $W_{REV,M,6M}$(5)= 0.76, **p = 0.039**, $W_{REV,M,14M}$(5)= 0.89, p = 0.346, $W_{REV,F,base}$(6)= 0.77, **p = 0.032**, $W_{REV,F,6M}$(5)= 0.89, p = 0.346, $W_{REV,F,14M}$(5)= 0.93, p = 0.617	Mixed effects + Fisher LSD	Time: F(1.538,12.30) = 4.99, **p = 0.033**; Sex: F(1,10) = 3.36, p = 0.097; Int: F(2,16) = 0.60, p = 0.561	M,base vs. M,6M **p=0.028**; M,base vs. M,14M p=0.587; M,6M vs. M,14M p=0.260; F,base vs. F,6M p=0.154; F,base vs. F,14M p=0.062; F,6M vs. F,14M p=0.521
S5a	Mean Arterial Pressure	**96.83 ± 14.99**	**5**	**95.33 ± 23.00**	**5**	**89.52 ± 25.92**	**5**	**98.00 ± 12.61**	**9**	**107.70 ± 9.90**	**8**	**73.80 ± 35.55**	**6**	$W_{REV,M,base}$(5)= 0.90, p = 0.430; $W_{REV,M,6M}$(5)= 0.67, **p = 0.004**, $W_{REV,M,14M}$(5)= 0.80, p = 0.085, $W_{REV,F,base}$(9)= 0.76, **p = 0.007**, $W_{REV,F,6M}$(8)= 0.93, p = 0.538, $W_{REV,F,14M}$(6)= 0.92, p = 0.481	Mixed effects + Fisher LSD	Time: F(1.681,17.68) = 5.64, **p = 0.014**; Sex: F(1,13) = 0.05, p = 0.833; Int: F(2,19) = 3.55, **p = 0.049**	M,base vs. M,6M p=0.422; M,base vs. M,14M p=0.062; M,6M vs. M,14M p=0.821; F,base vs. F,6M p=0.096; F,base vs. F,14M p=0.136; F,6M vs. F,14M **p=0.032**
S5b	Heart rate (bpm)	**604.50 ± 66.50**	**6**	**577.00 ± 38.00**	**5**	**549.00 ± 33.70**	**6**	**632.00 ± 97.20**	**6**	**594.00 ± 74.00**	**5**	**561.00 ± 35.50**	**5**	$W_{REV,M,base}$(6)= 0.96, p = 0.832; $W_{REV,M,6M}$(5)= 0.949, p = 0.729, $W_{REV,M,14M}$(6)= 0.74, **p = 0.015**, $W_{REV,F,base}$(6)= 0.97, p = 0.894, $W_{REV,F,6M}$(5)= 0.98, p = 0.938, $W_{REV,F,14M}$(5)= 0.98, p = 0.952	Mixed effects + Fisher LSD	Time: F(1.977,49.43) = 10.40, **p = 0.0002**; Sex: F(2,35) = 0.515, p = 0.602; Int: F(4,50) = 3.371, **p = 0.016**	M,base vs. M,6M p=0.236; M,base vs. M,14M p=0.435; M,6M vs. M,14M p=0.071; F,base vs. F,6M p=0.450; F,base vs. F,14M p=0.295; F,6M vs. F,14M p=0.055
S5c	End diastolic volume	34.00 ± 2.79	5	48.40 ± 4.46	5	43.80 ± 5.51	5	30.00 ± 0.97	6	39.00 ± 3.39	5	37.00 ± 2.61	5	$W_{REV,M,base}$(5)= 0.84, p = 0.177; $W_{REV,M,6M}$(5)= 0.84, p = 0.174, $W_{REV,M,14M}$(5)= 0.94, p = 0.655, $W_{REV,F,base}$(6)= 0.95, p = 0.740, $W_{REV,F,6M}$(5)= 0.93, p = 0.590, $W_{REV,F,14M}$(5)= 0.93, p = 0.585	Mixed effects + Fisher LSD	Time: F(1.665,12.48) = 10.65, **p = 0.003**; Sex: F(1,10) = 3.49, p = 0.092; Int: F(2,15) = 0.79, p = 0.472	M,base vs. M,6M p=0.057; M,base vs. M,14M p=0.091; M,6M vs. M,14M p=0.893; F,base vs. F,6M p=0.074; F,base vs. F,14M p=0.103; F,6M vs. F,14M p=0.268

Figure	Parameter	ND M	N	HSD M	N	REV M	N	ND F	N	HSD F	N	REV F	N	Shapiro-Wilk Test (Normality)	Comparison Test	Statistical Outcome (ANOVA statistic)	Statistical Outcome (post hoc)
6e	Media thickness	272.80 ± 11.79	5	400.20 ± 33.05	5	261.60 ± 20.70	5	307.30 ± 12.35	7	351.70 ± 28.32	9	293.00 ± 18.16	8	$W_{ND,M}$(5)= 0.90, p = 0.402; $W_{HSD,M}$(5)= 0.79, p = 0.073, $W_{REV,M}$(5)= 0.88, p = 0.302,<br>$W_{ND,F}$(7)= 0.95, p = 0.708, $W_{HSD,F}$(9)= 0.86, p = 0.089, $W_{REV,F}$(8)= 0.92, p = 0.392	Two-way ANOVA + Fisher LSD	Int: F(2,33) = 1.98, p = 0.154;<br>Sex: F(1,33) = 0.09, p = 0.767;<br>Diet: F(1,16) = 10.34, **p = 0.0003**	ND,M vs. HSD,M **p=0.002**; ND,M vs. Rev,M p=0.767; HSD,M vs. Rev,M **p=0.001**; ND,F vs. HSD,F p=0.146; ND,F vs. REV,F p=0.646; HSD,F vs. REV,F **p=0.050**
6f	Collagen deposition aorta (MTC)	**37.29 ± 36.70**	**6**	**32.05 ± 55.74**	**5**	**35.15 ± 50.21**	**5**	**38.74 ± 49.29**	**8**	**90.66 ± 63.84**	**9**	**34.03 ± 33.64**	**8**	$W_{ND,M}$(6)= 0.79, **p = 0.047**; $W_{HSD,M}$(5)= 0.90, p = 0.426, $W_{REV,M}$(5)= 0.96, p = 0.805,<br>$W_{ND,F}$(7)= 0.90, p = 0.296, $W_{HSD,F}$(9)= 0.94, p = 0.628, $W_{REV,F}$(8)= 0.91, p = 0.388	Two-way ANOVA + Fisher LSD	Int: F(2,35) = 4.42, **p = 0.019**;<br>Sex: F(1,35) = 4.21, **p = 0.048**;<br>Diet: F(2,35) = 3.11, p = 0.057	ND,M vs. HSD,M p=0.632; ND,M vs. Rev,M p=0.726; HSD,M vs. Rev,M p=0.903; ND,F vs. HSD,F **p=0.001**; ND,F vs. REV,F p=0.580; HSD,F vs. REV,F **p=0.0002**
6g	Elastin intensity	**24.75 ± 5.26**	**6**	**17.90 ± 6.38**	**5**	**25.62 ± 4.61**	**5**	**25.49 ± 6.83**	**8**	**26.99 ± 3.12**	**10**	**27.83 ± 7.20**	**8**	$W_{ND,M}$(6)= 0.86, p = 0.173; $W_{HSD,M}$(5)= 0.98, p = 0.959, $W_{REV,M}$(5)= 0.80, p = 0.075,<br>$W_{ND,F}$(8)= 0.94, p = 0.631, $W_{HSD,F}$(10)= 0.76, **p = 0.005**, $W_{REV,F}$(8)= 0.74, **p = 0.006**	Two-way ANOVA + Fisher LSD	Int: F(2,36) = 2.29., p = 0.116;<br>Sex: F(1,36) = 11.52, **p = 0.002**;<br>Diet: F(1,36) = 10.01, **p = 0.0004**	ND,M vs. HSD,M **p=0.014**; ND,M vs. Rev,M p=0.186; HSD,M vs. Rev,M **p=0.0006;** ND,F vs. HSD,F p=0.997; ND,F vs. REV,F **p=0.027**; HSD,F vs. REV,F **p=0.021**
6h	Tuft area (μm^2)	3618 ± 183	5	4581 ± 206	5	3707 ± 392	5	3349 ± 218	7	3693 ± 186	9	3806 ± 159	6	$W_{ND,M}$(5)= 0.80, p = 0.078; $W_{HSD,M}$(5)= 0.84, p = 0.158, $W_{REV,M}$(5)= 0.95, p = 0.752,<br>$W_{ND,F}$(7)= 0.92, p = 0.465, $W_{HSD,F}$(9)= 0.93, p = 0.528, $W_{REV,F}$(6)= 0.90, p = 0.385	Two-way ANOVA + Fisher LSD	Int: F(2,31) = 2.354, p = 0.112;<br>Sex: F(1,31) = 3.47, p = 0.072;<br>Diet: F(2,31) = 4.18, **p = 0.025**	ND,M vs. HSD,M **p=0.011**; ND,M vs. Rev,M p=0.805; HSD,M vs. Rev,M **p=0.020**; ND,F vs. HSD,F p=0.235; ND,F vs. REV,F p=0.155; HSD,F vs. REV,F p=0.706
6i	Glomerulosclerosis index	0.15 ± 0.06	6	0.09 ± 0.03	4	0.32 ± 0.16	5	0.63 ± 0.15	6	0.70 ± 0.12	9	0.69 ± 0.10	6	$W_{ND,M}$(6)= 0.80, p = 0.056; $W_{HSD,M}$(4)= 0.87, p = 0.292, $W_{REV,M}$(5)= 0.78, p = 0.052,<br>$W_{ND,F}$(6)= 0.95, p = 0.761, $W_{HSD,F}$(9)= 0.96, p = 0.823, $W_{REV,F}$(6)= 0.98, p = 0.939	Two-way ANOVA + Fisher LSD	Int: F(2,30) = 0.47, p = 0.628;<br>Sex: F(1,30) = 23.22, **p < 0.0001**;<br>Diet: F(2,30) = 0.59, p = 0.563	ND,M vs. HSD,M p=0.752; ND,M vs. Rev,M p=0.328; HSD,M vs. Rev,M p=0.238; ND,F vs. HSD,F p=0.674; ND,F vs. REV,F p=0.728; HSD,F vs. REV,F p=0.967
6k	Vessel wall thickness (10-40μm)	4.46 ± 0.37	5	4.66 ± 0.25	5	4.00 ± 0.33	5	3.93 ± 0.30	7	5.37 ± 0.25	9	4.35 ± 0.20	6	$W_{ND,M}$(5)= 0.94, p = 0.677; $W_{HSD,M}$(5)= 0.96, p = 0.794, $W_{REV,M}$(5)= 0.96, p = 0.810,<br>$W_{ND,F}$(7)= 0.95, p = 0.759, $W_{HSD,F}$(9)= 0.96, p = 0.749, $W_{REV,F}$(6)= 0.96, p = 0.880	Two-way ANOVA + Fisher LSD	Int: F(2,31) = 2.48, p = 0.100;<br>Sex: F(1,31) = 0.56, p = 0.459;<br>Diet: F(2,31) = 5.64, **p = 0.008**	ND,M vs. HSD,M p=0.667; ND,M vs. Rev,M p=0.305; HSD,M vs. Rev,M p=0.149; ND,F vs. HSD,F **p=0.0003**; ND,F vs. REV,F p=0.296; HSD,F vs. REV,F **p=0.010**
S6a	Aorta wall thickness	**65.97 ± 18.40**	**6**	**93.25 ± 24.02**	**5**	**64.10 ± 34.83**	**5**	**79.42 ± 22.05**	**8**	**81.66 ± 27.28**	**9**	**76.07 ± 30.85**	**8**	$W_{ND,M}$(6)= 0.79, p = 0.052; $W_{HSD,M}$(5)= 0.91, p = 0.441, $W_{REV,M}$(5)= 0.90, p = 0.383,<br>$W_{ND,F}$(8)= 0.94, p = 0.609, $W_{HSD,F}$(9)= 0.80, **p = 0.020**, $W_{REV,F}$(8)= 0.97, p = 0.902	Two-way ANOVA + Fisher LSD	Int: F(2,35) = 1.80, p = 0.180;<br>Sex: F(1,35) = 1.89, p = 0.178;<br>Diet: F(2,35) = 4.69, **p = 0.016**	ND,M vs. HSD,M **p=0.006**; ND,M vs. Rev,M p=0.750; HSD,M vs. Rev,M **p=0.019**; ND,F vs. HSD,F p=0.519; ND,F vs. REV,F p=0.655; HSD,F vs. REV,F p=0.272
S6b	Aorta SMA expression	**65.81 ± 22.94**	**6**	**47.13 ± 10.53**	**5**	**51.50 ± 19.41**	**5**	**63.56 ± 7.67**	**7**	**64.80 ± 6.59**	**9**	**65.64 ± 6.33**	**8**	$W_{ND,M}$(6)= 0.92, p = 0.553; $W_{HSD,M}$(5)= 0.94, p = 0.651, $W_{REV,M}$(5)= 0.85, p = 0.189,<br>$W_{ND,F}$(7)= 0.95, p = 0.694, $W_{HSD,F}$(9)= 0.93, p = 0.436, $W_{REV,F}$(8)= 0.79, **p = 0.021**	Two-way ANOVA + Fisher LSD	Int: F(2,34) = 2.47, p = 0.100;<br>Sex: F(1,34) = 10.21, **p = 0.003**;<br>Diet: F(2,34) = 2.63, p = 0.087	ND,M vs. HSD,M **p=0.006**; ND,M vs. Rev,M p=0.208; HSD,M vs. Rev,M p=0.125; ND,F vs. HSD,F p=0.955; ND,F vs. REV,F p=0.959; HSD,F vs. REV,F p=0.997
S6c	Aorta OPN expression	5.78 ± 0.33	6	8.99 ± 0.59	5	8.03 ± 0.43	5	7.44 ± 0.44	10	6.67 ± 0.25	8	8.55 ± 1.82	8	$W_{ND,M}$(6)= 0.92, p = 0.526; $W_{HSD,M}$(5)= 0.84, p = 0.168, $W_{REV,M}$(5)= 0.91, p = 0.493,<br>$W_{ND,F}$(10)= 0.93, p = 0.401, $W_{HSD,F}$(8)= 0.96, p = 0.763, $W_{REV,F}$(8)= 0.94, p = 0.628	Two-way ANOVA + Fisher LSD	Int: F(2,36) = 2.26, p = 0.119;<br>Sex: F(1,36) = 0.003, p = 0.951;<br>Diet: F(1,36) = 1.70, p = 0.196	ND,M vs. HSD,M **p=0.039**; ND,M vs. Rev,M p=0.141; HSD,M vs. Rev,M p=0.545; ND,F vs. HSD,F p=0.517; ND,F vs. REV,F p=0.354; HSD,F vs. REV,F p=0.139
S6d	Aorta SMA:OPN ratio	**4.30 ± 0.40**	**6**	**2.20 ± 0.87**	**5**	**2.37 ± 0.51**	**5**	**1.93 ± 0.43**	**10**	**2.15 ± 0.30**	**8**	**1.94 ± 2.35**	**8**	$W_{ND,M}$(6)= 0.93, p = 0.597; $W_{HSD,M}$(5)= 0.93, p = 0.610, $W_{REV,M}$(5)= 0.87, p = 0.276,<br>$W_{ND,F}$(10)= 0.791, **p = 0.011**, $W_{HSD,F}$(8)= 0.93, p = 0.529, $W_{REV,F}$(8)= 0.85, p = 0.100	Two-way ANOVA + Fisher LSD	Int: F(2,36) = 15.81, **p < 0.0001**;<br>Sex: F(1,36) = 16.20, **p = 0.0003**;<br>Diet: F(2,36) = 9.46, **p = 0.0005**	ND,M vs. HSD,M **p<0.0001**; ND,M vs. Rev,M **p<0.0001**; HSD,M vs. Rev,M p=0.866; ND,F vs. HSD,F p=0.640; ND,F vs. REV,F p=0.190; HSD,F vs. REV,F p=0.419
S7a	Renin mRNA expression	1.00 ± 0.15	6	0.42 ± 0.14	5	1.50 ± 0.10	4	1.00 ± 0.15	5	0.18 ± 0.02	9	2.61 ± 0.61	6	$W_{ND,M}$(6)= 0.85, p = 0.171; $W_{HSD,M}$(5)= 0.85, p = 0.198, $W_{REV,M}$(4)= 0.90, p = 0.426,<br>$W_{ND,F}$(5)= 0.95, p = 0.753, $W_{HSD,F}$(9)= 0.96, p = 0.768, $W_{REV,F}$(6)= 0.98, p = 0.932	Two-way ANOVA + Fisher LSD	Int: F(2,29) = 3.12, p = 0.059;<br>Sex: F(1,29) = 1.57, p = 0.221;<br>Diet: F(2,29) = 19.39, **p < 0.0001**	ND,M vs. HSD,M p=0.159; ND,M vs. Rev,M p=0.252; HSD,M vs. Rev,M **p=0.0215**; ND,F vs. HSD,F **p=0.033**; ND,F vs. REV,F **p=0.0004**; HSD,F vs. REV,F **p<0.0001**
S7b	Aqp2 mRNA expression	0.91 ± 0.13	6	0.91 ± 0.06	5	1.05 ± 0.14	5	1.00 ± 0.25	5	0.65 ± 0.04	9	0.84 ± 0.10	6	$W_{ND,M}$(6)= 0.90, p = 0.396; $W_{HSD,M}$(5)= 0.81, p = 0.092, $W_{REV,M}$(5)= 0.85, p = 0.207,<br>$W_{ND,F}$(5)= 0.95, p = 0.741, $W_{HSD,F}$(9)= 0.91, p = 0.313, $W_{REV,F}$(5)= 0.81, p = 0.101	Two-way ANOVA + Fisher LSD	Int: F(2,29) = 1.16, p = 0.327;<br>Sex: F(1,29) = 1.633, p = 0.211;<br>Diet: F(2,29) = 1.34, p = 0.277	ND,M vs. HSD,M p=0.997; ND,M vs. Rev,M p=0.440; HSD,M vs. Rev,M p=0.457; ND,F vs. HSD,F **p=0.042**; ND,F vs. REV,F p=0.401; HSD,F vs. REV,F p=0.253
S7c	CD86 mRNA expression	**0.94 ± 0.61**	**6**	**0.91 ± 0.55**	**5**	**0.72 ± 0.60**	**3**	**0.82 ± 0.47**	**5**	**1.71 ± 1.00**	**9**	**1.24 ± 0.50**	**5**	$W_{ND,M}$(6)= 0.90, p = 0.348; $W_{HSD,M}$(5)= 0.90, p = 0.408, $W_{REV,M}$(3)= 0.81, p = 0.135,<br>$W_{ND,F}$(5)= 0.646, **p = 0.002**, $W_{HSD,F}$(9)= 0.91, p = 0.307, $W_{REV,F}$(5)= 0.96, p = 0.792	Two-way ANOVA + Fisher LSD	Int: F(2,27) = 1.46, p = 0.250;<br>Sex: F(1,27) = 4.01, p = 0.055;<br>Diet: F(2,27) = 1.93, p = 0.165	ND,M vs. HSD,M p=0.913; ND,M vs. Rev,M p=0.743; HSD,M vs. Rev,M p=0.683; ND,F vs. HSD,F **p=0.014**; ND,F vs. REV,F p=0.381; HSD,F vs. REV,F p=0.115
S7d	Lcn2 mRNA expression	**1.00 ± 0.34**	**6**	**0.85 ± 0.23**	**5**	**0.80 ± 1.29**	**4**	**0.89 ± 0.54**	**5**	**1.32 ± 1.19**	**9**	**3.12 ± 2.17**	**4**	$W_{ND,M}$(6)= 0.89, p = 0.297; $W_{HSD,M}$(5)= 0.80, p = 0.075, $W_{REV,M}$(4)= 0.72, **p = 0.019**,<br>$W_{ND,F}$(5)= 0.90, p = 0.386, $W_{HSD,F}$(9)= 0.702, **p = 0.002**, $W_{REV,F}$(4)= 0.77, p = 0.056	Two-way ANOVA + Fisher LSD	Int: F(2,27) = 2.06, p = 0.148;<br>Sex: F(1,27) = 8.68, **p = 0.007**;<br>Diet: F(2,27) = 2.95, p = 0.070	ND,M vs. HSD,M p=0.668; ND,M vs. Rev,M p=0.721; HSD,M vs. Rev,M p=0.482; ND,F vs. HSD,F p=0.085; ND,F vs. REV,F **p=0.006**; HSD,F vs. REV,F p=0.103
S9	Vessel wall thickness (40-80μm)	6.11 ± 0.45	5	6.76 ± 0.44	5	6.06 ± 0.68	5	5.94 ± 0.50	7	7.84 ± 0.38	9	8.25 ± 0.91	6	$W_{ND,M}$(5)= 0.94, p = 0.651; $W_{HSD,M}$(5)= 0.89, p = 0.349, $W_{REV,M}$(5)= 0.96, p = 0.784,<br>$W_{ND,F}$(7)= 0.95, p = 0.739, $W_{HSD,F}$(9)= 0.84, p = 0.062, $W_{REV,F}$(6)= 0.98, p = 0.929	Two-way ANOVA + Fisher LSD	Int: F(2,31) = 1.94, p = 0.161;<br>Sex: F(1,31) = 4.63, **p = 0.039**;<br>Diet: F(2,31) = 2.85, p = 0.073	ND,M vs. HSD,M p=0.477; ND,M vs. Rev,M p=0.960; HSD,M vs. Rev,M p=0.447; ND,F vs. HSD,F **p=0.013**; ND,F vs. REV,F **p=0.007**; HSD,F vs. REV,F p=0.587

Figure	Parameter	ND 0	N	ND 6	N	ND 14	N	HSD 0	N	HSD 6	N	HSD 14	N	Shapiro-Wilk Test (Normality)	Comparison Test	Statistical Outcome (ANOVA statistic)	Statistical Outcome (post hoc)
S2a	Heart rate - male (bpm)	**610.00 ± 116.80**	**6**	**600.00 ± 36.00**	**5**	**570.00 ± 59.50**	**5**	**588.50 ± 76.80**	**6**	**628.00 ± 111.50**	**5**	**527.50 ± 80.00**	**6**	$W_{ND,M,base}$(6)= 0.97, p = 0.866; $W_{ND,M,6M}$(5)= 0.95, p = 0.753, $W_{ND,M,14M}$(5)= 0.97, p = 0.900,<br>$W_{HSD,M,base}$(6)= 0.77, **p = 0.030**, $W_{HSD,M,6M}$(5)= 0.91, p = 0.454, $W_{HSD,M,14M}$(6)= 0.97, p = 0.888	Mixed effects + Fisher LSD	Time: F(1.978,14.83) = 6.479, **p = 0.010**;<br>Diet: F(1,12) = 1.225, p = 0.290;<br>Int: F(2,15) = 1.953, p = 0.176	M,ND,base vs. M,ND,6M p=0.730; M,ND,base vs. M,ND,14M p=0.576; M,ND,6M vs. M,ND,14M p=0.487; M,HSD,base vs. M,HSD,6M p=0.902; M,HSD,base vs. M,HSD,14M **p=0.014**; M,HSD,6M vs. M,HSD,14M **p=0.049**;<br>ND,base vs. HSD,base p=0.827; ND,6M vs. HSD,6M p=0.589; ND,14M vs. HSD,14M **p=0.046**
S2b	Heart rate - female (bpm)	596.70 ± 18.65	6	605.70 ± 25.69	6	557.50 ± 16.84	6	592.00 ± 15.89	6	569.00 ± 15.72	5	543.00 ± 16.00	5	$W_{ND,F,base}$(6)= 0.93, p = 0.615; $W_{ND,F,6M}$(6)= 0.93, p = 0.605, $W_{ND,F,14M}$(6)= 0.95, p = 0.728,<br>$W_{HSD,F,base}$(6)= 0.81, p = 0.068, $W_{HSD,F,6M}$(5)= 0.98, p = 0.913, $W_{HSD,F,14M}$(5)= 0.85, p = 0.194	Mixed effects + Fisher LSD	Time: F(1.640,14.76) = 4.229, **p = 0.042**;<br>Diet: F(1,10) = 0.831, p = 0.384;<br>Int: F(2,18) = 0.478, p = 0.628	F,ND,base vs. F,ND,6M p=0.726; F,ND,base vs. F,ND,14M p=0.162; F,ND,6M vs. F,ND,14M p=0.068; F,HSD,base vs. F,HSD,6M p=0.564; F,HSD,base vs. F,HSD,14M p=0.193; F,HSD,6M vs. F,HSD,14M **p=0.014**;<br>ND,base vs. HSD,base p=0.853; ND,6M vs. HSD,6M p=0.258; ND,14M vs. HSD,14M p=0.548

Figure	Parameter	HSD M 0	N	HSD M 6	N	HSD M 14	N	HSD F 0	N	HSD F 6	N	HSD F 14	N	Shapiro-Wilk Test (Normality)	Comparison Test	Statistical Outcome (ANOVA statistic)	Statistical Outcome (post hoc)
S2c	Stroke volume (μL)	22.17 ± 2.61	6	26.80 ± 3.80	5	34.60 ± 1.94	5	22.67 ± 0.33	6	26.60 ± 2.54	5	24.25 ± 2.10	4	$W_{HSD,M,base}$(6)= 0.95, p = 0.709; $W_{HSD,M,6M}$(5)= 0.91, p = 0.455, $W_{HSD,M,14M}$(5)= 0.95, p = 0.747,<br>$W_{HSD,F,base}$(6)= 0.82, p = 0.091, $W_{HSD,F,6M}$(5)= 0.94, p = 0.635, $W_{HSD,F,14M}$(4)= 0.94, p = 0.650	Mixed effects + Fisher LSD	Time: F(1.715,21.44) = 4.347, **p = 0.031**;<br>Sex: F(1,25) = 2.830, p = 0.105;<br>Int: F(2,25) = 2.92, p = 0.072	M,base vs. M,6M p=0.301; M,base vs. M,14M **p=0.026**; M,6M vs. M,14M p=0.160; F,base vs. F,6M p=0.156; F,base vs. F,14M p=0.457; F,6M vs. F,14M p=0.939, M,base vs. F,base p=0.857; M,6M vs. F,6M p=0.966; M,14M vs. F,14M **p=0.009**
S2d	End-diastolic volume (μL)	31.50 ± 2.32	6	44.00 ± 4.87	5	52.80 ± 3.89	5	30.00 ± 0.93	6	37.00 ± 1.84	5	34.00 ± 2.74	4	$W_{HSD,M,base}$(6)= 0.88, p = 0.247; $W_{HSD,M,6M}$(5)= 0.97, p = 0.842, $W_{HSD,M,14M}$(5)= 0.92, p = 0.517,<br>$W_{HSD,F,base}$(6)= 0.81, p = 0.079, $W_{HSD,F,6M}$(5)= 0.79, p = 0.062, $W_{HSD,F,14M}$(4)= 0.82, p = 0.139	Mixed effects + Fisher LSD	Time: F(1.491,10.43) = 12.81, **p = 0.003**;<br>Sex: F(1,11) = 11.42, **p = 0.006**;<br>Int: F(2,14) = 4.95, **p = 0.024**	M,base vs. M,6M p=0.093; M,base vs. M,14M **p=0.042**; M,6M vs. M,14M p=0.157; F,base vs. F,6M **p=0.007**; F,base vs. F,14M p=0.160; F,6M vs. F,14M p=0.647, M,base vs. F,base p=0.569; M,6M vs. F,6M p=0.235; M,14M vs. F,14M **p=0.006**

Table S3. Left ventricle gene expression.

	male					female				
	ND		HSD			ND		HSD		
	mean	SD	mean	SD	P-value	mean	SD	mean	SD	P-value
Tgfb1	1,4437	0,2201	1,3780	0,0773	0,5435					
Fabp3	0,8171	0,1346	0,7254	0,0871	0,3110	0,7189	0,1552	0,7877	0,1713	0,3530
Nppb	1,1107	0,3385	1,8722	1,0120	**0,0338**	0,4911	0,1951	1,0085	0,5604	**0,0761**
Col3a1	0,9159	0,1652	0,7855	0,0649	0,5696	0,8714	0,4408	0,9887	0,4916	0,5359
Tnnt2	1,7335	0,3221	1,4419	0,1095	**0,0325**	0,9681	0,1562	1,1013	0,2041	0,2205
Fib	0,7439	0,1891	0,8142	0,1266	0,4976					
Col1a1	0,8818	0,1307	0,8684	0,1694	0,9365	0,8158	0,2402	0,8530	0,3925	0,9394
Acta2	0,2585	0,0843	0,4106	0,2961	0,5619	0,7191	0,3187	0,9962	0,2937	**0,0034**
Tnni3	1,2676	0,2549	1,4158	0,3338	0,4247					
Pdgfra	1,3513	0,4306	1,3980	0,0974	0,8189					
Pdgfrb	0,9246	0,1653	0,8126	0,0802	0,2016					
Anxa1	1,1077	0,1677	1,2573	0,1653	0,1502	0,7504	0,1078	0,8950	0,2077	0,0947

Acta2 – Actin Alpha 2, Smooth Muscle, Anxa1 – Annexin A1, Col1a1 – Collagen Type I Alpha 1 Chain, Col3a1 – Collagen Type III Alpha 1 Chain, Fabp3 – Fatty Acid Binding Protein 3, Fib –Fibrinogen, Nppb – Natriuretic Peptide B, Pdgfra – Platelet-Derived Growth Factor Receptor Alpha, Pdgfrb – Platelet-Derived Growth Factor Receptor Beta, Tgfb1 – Transforming Growth Factor Beta 1, Tnni3 – Troponin I3, Cardiac Type, Tnnt2 – Troponin T2, Cardiac Type

Table S4. Right ventricle gene expression.

	male					female				
	ND		HSD			ND		HSD		
	mean	SD	mean	SD	P-value	mean	SD	mean	SD	P-value
Col1a1	1,0215	0,2698	0,7788	0,2974	0,1648	0,7255	0,3152	0,9074	0,2499	0,1971
Col3a	1,1634	0,3670	0,8465	0,5034	0,1871	0,8525	0,3904	0,9414	0,3185	0,6395
Fabp3	1,1721	0,1705	0,9699	0,1125	**0,0500**	0,8866	0,2538	0,7570	0,1425	0,1591
Anxa1	1,0355	0,2437	0,8728	0,1809	0,4248	1,0709	0,5176	0,9481	0,2015	0,4522
Nppb	0,9276	0,2478	1,2463	0,5129	0,2765	0,6700	0,4889	1,1440	0,5397	**0,0500**
Tnnt2	1,1208	0,2790	0,8993	0,2297	0,1213	1,0590	0,2527	0,8527	0,1571	**0,0476**
Acta2	0,8380	0,3389	1,1310	0,5566	0,1643	0,6275	0,1689	1,0268	0,2993	**0,0226**

Acta2 – Actin Alpha 2, Smooth Muscle, Anxa1 – Annexin A1, Col1a1 – Collagen Type I Alpha 1 Chain, Col3a1 – Collagen Type III Alpha 1 Chain, Fabp3 – Fatty Acid Binding Protein 3, Fib –Fibrinogen, Nppb – Natriuretic Peptide BTnnt2 – Troponin T2, Cardiac Type

Table S5. Mesenteric artery gene expression.

	male					female				
	ND		HSD			ND		HSD		
	mean	SD	mean	SD	P-value	mean	SD	mean	SD	P-value
Egfr1	1,5895	0,8873	0,9898	0,4810	0,2607	1,9310	1,1714	0,8709	0,3431	**0,0281**
Vwf	2,0068	1,4319	2,6138	0,3895	0,2548	0,9471	0,3580	0,8513	0,1726	0,8378
Adra1	1,7300	0,7763	1,2830	0,2923	0,2901	1,2595	0,4726	1,4024	0,7427	0,6909
Sphk1	3,2925	1,3566	2,2455	0,9315	0,1065	0,7197	0,1989	1,3267	0,9493	0,2683
Klf4	3,1345	0,5785	2,5090	0,2018	**0,0091**	0,7353	0,1047	1,1126	0,6426	0,4355
Nos3	1,1643	0,4852	0,8933	0,3158	0,8586	3,5113	3,0790	3,5841	2,9939	0,9557
Acta2	2,6374	0,5705	2,4656	0,5055	0,1685	0,9245	0,2552	0,9361	0,1310	0,9741
Agt1r	3,0100	1,1291	2,4682	0,3156	0,2235	1,0966	0,4100	1,1648	0,3956	0,8561
C3	0,5463	0,4606	0,2377	0,1046	0,4650	0,7818	0,3499	1,4869	0,9058	**0,0500**
Vim	0,5840	0,4853	0,1797	0,0584	0,2191	0,6372	0,2346	0,7888	0,6252	0,5654
Chrm1	1,2221	0,1888	1,1094	0,7499	0,7138	0,2140	0,0951	0,4633	0,2425	0,4536
Chrm3	0,0018	0,0031	0,0003	0,0002	**0,0018**	0,0790	0,0996	0,1424	0,2021	0,6369

Acta2 – Actin Alpha 2, Smooth Muscle, Adra1 –Adrenergic Receptor Alpha 1, Agt1r – Angiotensin II Type 1 Receptor, C3 – Complement Component 3, Chrm1 – Cholinergic Receptor Muscarinic 1, Chrm3 – Cholinergic Receptor Muscarinic 3, Egfr1 – Epidermal Growth Factor Receptor 1, Nos3 – Endothelial Nitric Oxide Synthase, Klf4 – Kruppel-Like Factor 4, Sphk1 – Sphingosine Kinase 1, Vim – Vimentin, Vwf – Von Willebrand Factor

Table S6. Kidney gene expression.

	male					female				
	ND		HSD			ND		HSD		
	mean	SD	mean	SD	P-value	mean	SD	mean	SD	P-value
Renin	1,0145	0,3619	0,4276	0,3121	**0,0014**	1,0360	0,3150	0,1870	0,0504	**0,0000**
Agt2r	1,0078	0,5811	1,5208	0,9822	0,3339	0,8649	1,5690	0,1020	0,0433	0,1252
Slc9a3	0,8740	0,0854	0,9169	0,2025	0,6173	1,0210	0,1586	0,8696	0,1161	0,0653
Aqp2	0,8306	0,2962	0,8297	0,1315	0,9971	1,4828	0,8270	0,9567	0,1771	**0,0310**
Crip1	0,7447	0,1942	1,1107	0,2696	**0,0161**	0,7434	0,1801	0,8530	0,2526	0,4042
Cited4	0,8804	0,0676	1,0950	0,6090	0,2504	1,4642	0,2227	1,0075	0,1800	**0,0114**
Tnfa	3,0153	3,2537	0,5684	0,5974	**0,0217**	0,3408	0,1295	0,6449	0,4089	0,7413
Ifng	1,9200	1,5800	2,0900	1,1670	0,7669	0,3938	0,2492	0,6296	0,4519	0,8588
Tgfb1	1,2476	0,1252	1,2519	0,2092	0,9780	0,7554	0,1400	1,0434	0,3639	0,1194
Cd86	0,8290	0,2988	0,8554	0,2781	0,8782	0,4069	0,1284	0,6885	0,3215	0,0860
Il6	0,0719	0,0282	0,1827	0,1680	0,5429	0,2173	0,0920	0,6920	0,3775	0,1668
Lcn2	0,7208	0,1606	0,5679	0,0989	0,5099	0,5438	0,1700	0,9711	0,5802	**0,0547**
Ank1	0,9767	0,1545	1,0778	0,2414	0,3056	1,2178	0,1841	0,9764	0,0734	**0,0128**
Sgk1	0,5415	0,0570	0,8036	0,1827	**0,0522**	1,1767	0,3195	0,9942	0,2152	0,1347
Aqp1	0,8668	0,1880	0,9205	0,2587	0,6866	1,3262	0,2759	1,0248	0,1717	**0,0210**
Scnn1	0,9922	0,1728	0,9797	0,2205	0,9328	1,4370	0,2481	1,2845	0,2842	0,2724
Agt1r	0,8600	0,1373	0,9263	0,2003	0,5314	1,1651	0,1879	0,9491	0,1695	**0,0358**

Ank1 – Ankyrin 1, Aqp1 – Aquaporin 1, Aqp2 – Aquaporin 2, Agt1r – Angiotensin II Type 1 Receptor, Agt2r – Angiotensin II Type 2 Receptor, CD86 – Cluster of Differentiation 86, Cited4 – Cbp/P300-Interacting Transactivator with Glu/Asp-Rich Carboxy-Terminal Domain 4, Crip1 – Cysteine-Rich Protein 1, Scnn1 – Sodium Channel Epithelial 1, Ifng – Interferon Gamma, Il6 – Interleukin 6, Lcn2 – Lipocalin 2, Slc9a3 – Sodium-Hydrogen Exchanger 3, Ren – Renin, Sgk1 – Serum/Glucocorticoid-Regulated Kinase 1, Tgfb1 – Transforming Growth Factor Beta 1, Tnfa – Tumor necrosis factor alpha

Figure S1. Effect of high salt diet on body weight, food and water intake in male and female mice.

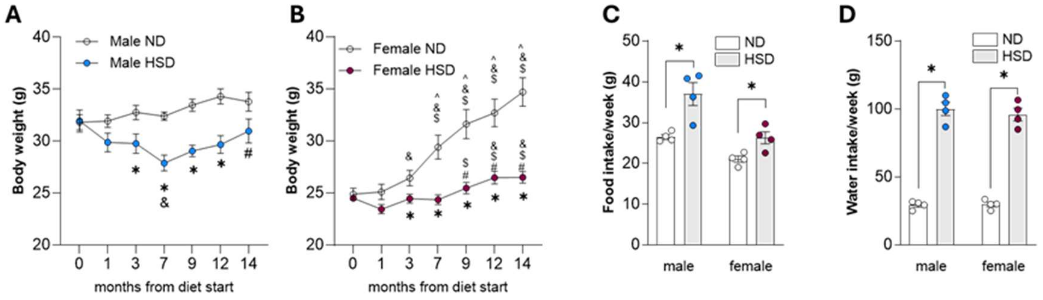

(A) Body weight development in male mice in response to 14-months of normal diet (ND) or high salt diet (HSD) feeding. **(B)** Body weight development in female mice in response to 14-months of ND or HSD feeding. **(C)** Average weekly food intake over 4 weeks of male and female HSD mice compared to their respective ND controls. **(D)** Average weekly water intake over 4 weeks of male and female HSD mice compared to their respective ND controls.
*Values are given as mean ± SEM. In panels A-B, * denotes P<0.05 compared to ND; & denotes P<0.05 compared to baseline of same group, $ denotes P<0.05 compared to 1-month timepoint of same group, ^ denotes P<0.05 compared to 3-months timepoint of same group, # denotes P<0.05 compared to 7-months timepoint of same group after RM-ANOVA and Fisher's LSD post-hoc testing. In panels C-D, * denotes P<0.05 after 2-Way ANOVA and Fisher's LSD post-hoc testing.*

Figure S2. High salt diet induces alterations in cardiac function in male but not female mice.

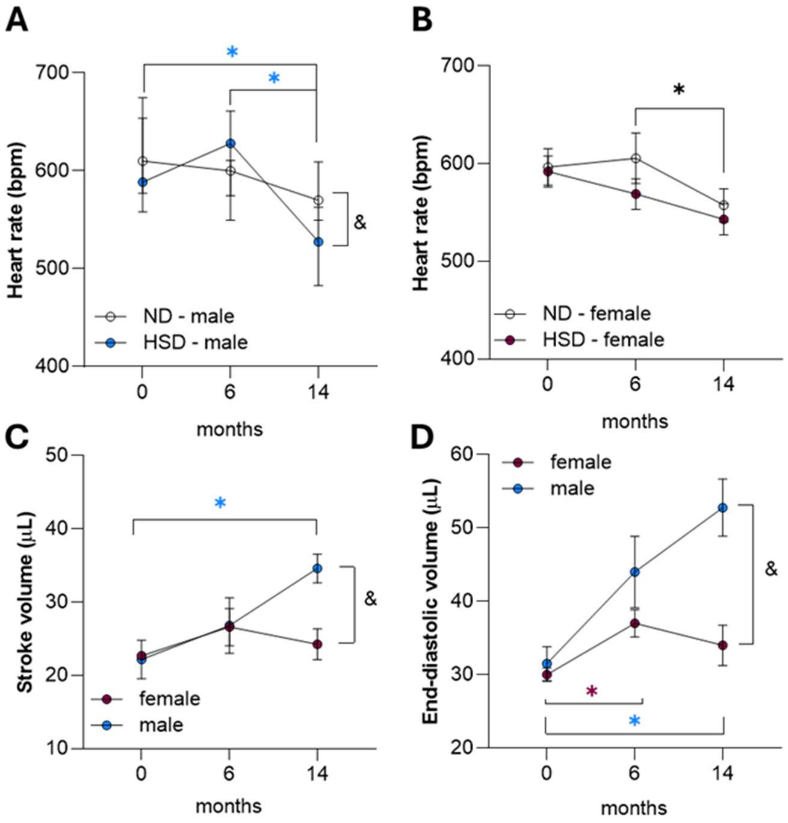

Longitudinal heart rate recording showing HR lowering in **(A)** male and **(B)** female mice after high salt diet (HSD) and normal diet (ND) interventions. **(C)** Magnetic resonance imaging-based assessment of cardiac function showing changes in stroke volume and **(D)** end-diastolic volume in HSD mice.

*In panel A , values are given as median ± IQR; in panels B to D, values are given as mean ± SEM. * denotes P<0.05 after RM-ANOVA and Fisher's LSD post-hoc testing.*

Figure S3. High salt diet induces alterations of aortic smooth muscle cells in male but not female mice.

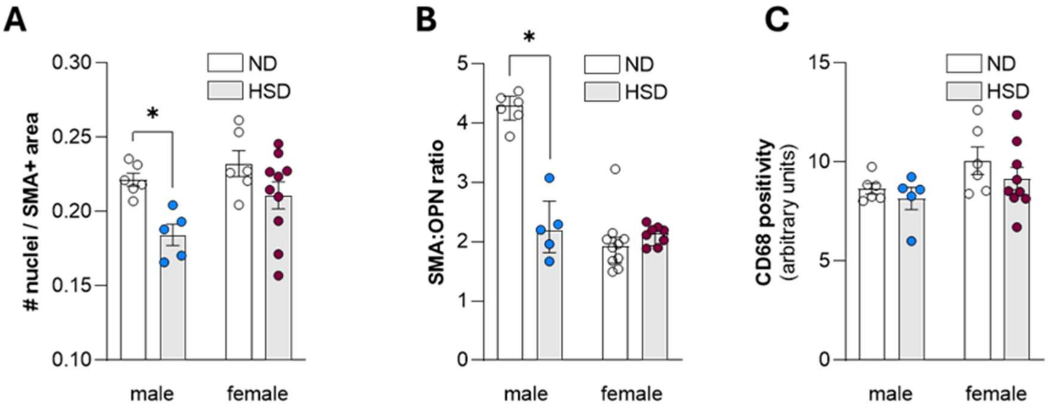

(A) Aorta nuclei quantification showing less nuclei per smooth muscle actin (SMA)+ area in male high salt diet (HSD) fed mice. **(B)** Aorta SMA-to-osteopontin (OPN) ratio is lower in male HSD mice compared to normal diet (ND) controls. **(C)** Quantification of immunofluorescence aorta staining showing similar CD68+ cells in male and female HSD mice compared to their respective ND controls.

*In panels A and C, values are given as mean ± SEM; in panel B, values are given as median ± IQR. * denotes P<0.05 after 2-Way ANOVA and Fisher's LSD post-hoc testing.*

Figure S4. High dietary salt intake impairs mesenteric artery function.

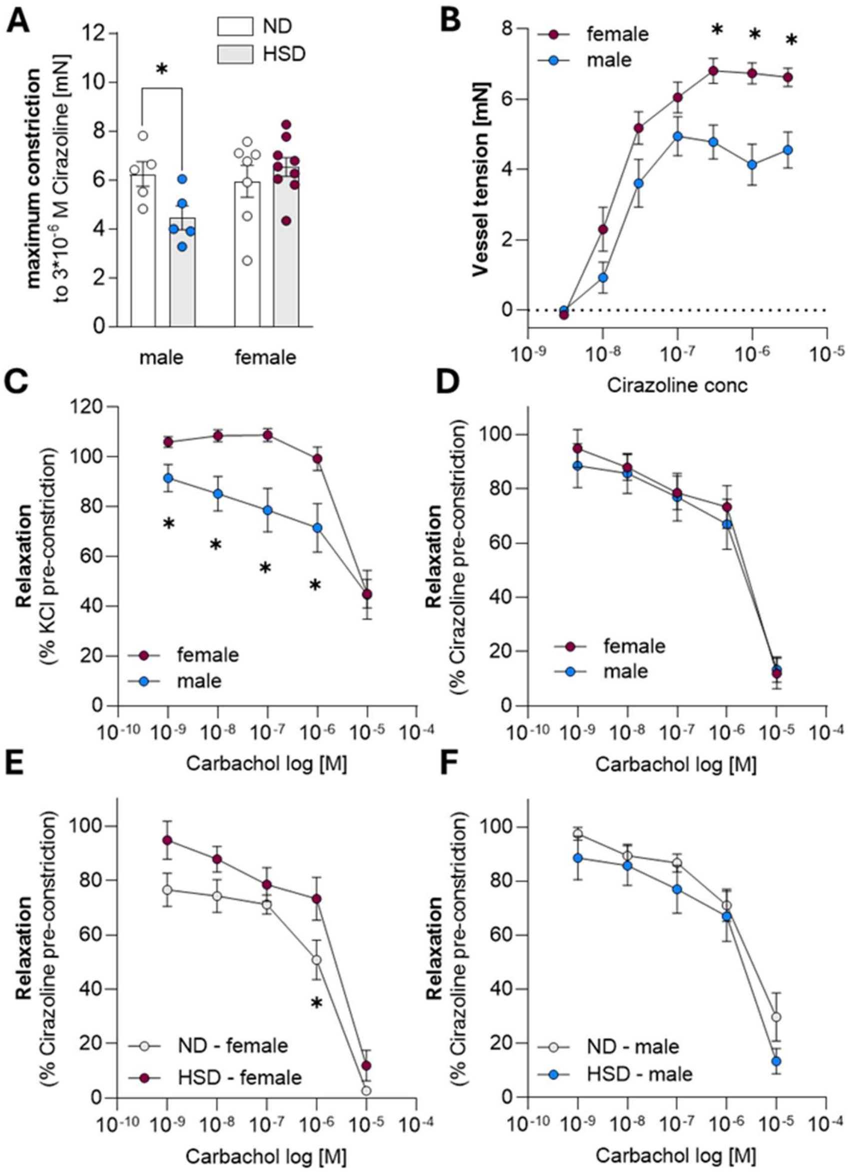

Wire myography used to assess vascular functionality showing **(A)** lower maximum constriction to cirazoline (3x10^{-6} M) in male mice on high salt diet (HSD) compared to control diet (ND). Vessel tension was lower in male compared to female HSD mice in response to increasing concentrations of **(B)** the alpha 1 adrenergic receptor agonist Cirazoline and **(C)** the parasympathomimetic carbachol after potassium chloride (KCl) preconstruction and **(D)** cirazoline pre-constriction. **(E)** Female HSD mice showed higher vasorelaxation in response to carbachol compared to ND controls, whereas **(F)** responses of male HSD mice did not differ from those of ND controls.

*Values are given as mean ± SEM. In panel A, * denotes P<0.05 after 2-Way ANOVA and Fisher's LSD post-hoc testing. In panels B to F, * denotes P<0.05 after RM-ANOVA and Fisher's LSD post-hoc testing.*

Figure S5. Normalizing dietary salt intake lowers blood pressure in female mice.

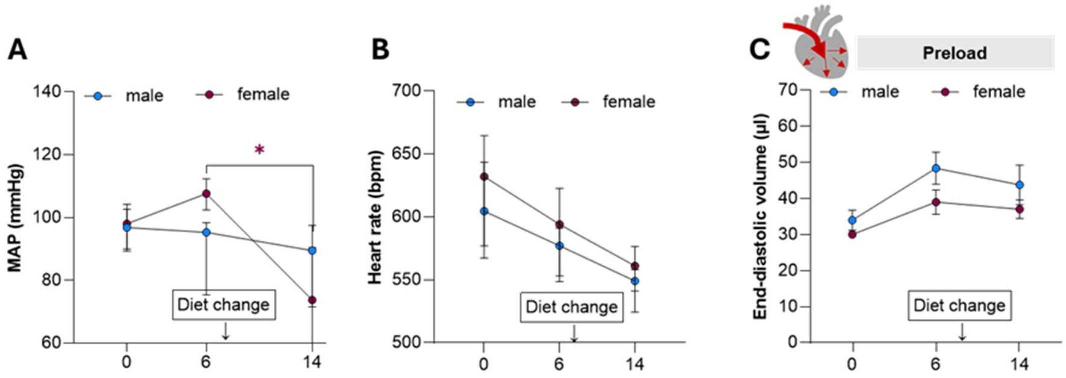

Lowering dietary salt content **(A)** reduces elevated mean arterial pressure (MAP), **(B)** lowers heart rate, and **(C)** has no effect on end-diastolic volume.

In panels A and B, values given as median ± IQR; in panel C, values are given as mean ± SEM.
** denotes P<0.05 after RM-ANOVA and Fisher's LSD post-hoc testing.*

Figure S6. Lowering high dietary salt intake affects aorta morphology and cell content.

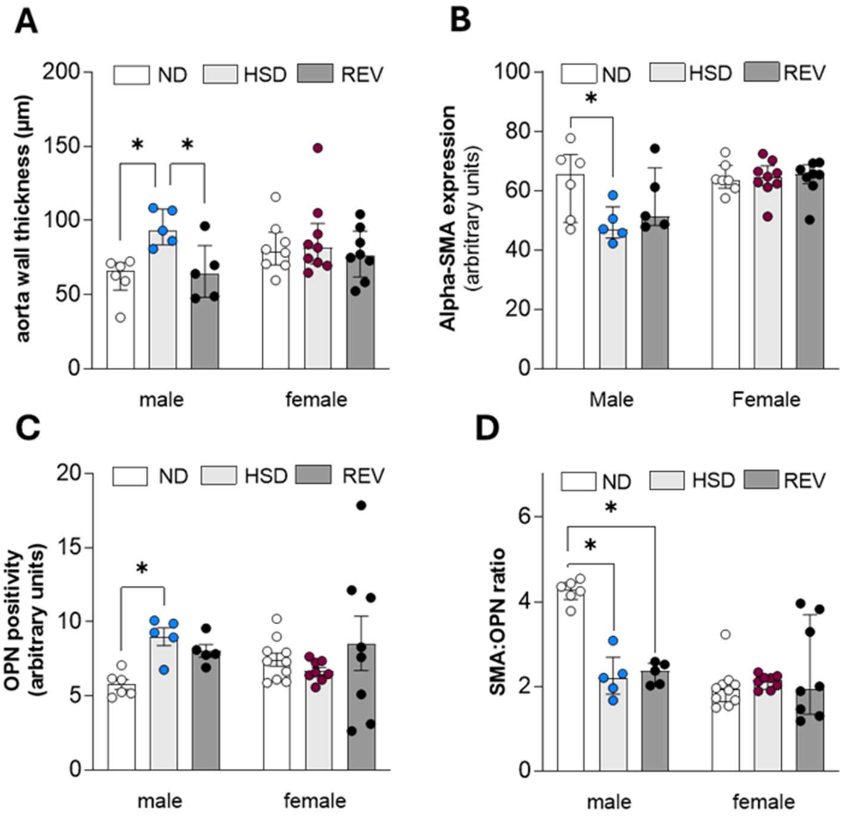

(A) Quantification of H&E staining of the aorta showing increased aortic wall thickness in male high salt diet (HSD) mice that was absent in the group with diet reversal (REV). **(B)** Quantification of immunofluorescence smooth muscle actin (SMA) staining showing less SMA+ cells in male HSD mice. SMA+ cells in mice of the REV group were lower than normal diet (ND) controls but higher compared to HSD mice. **(C)** Quantification of immunofluorescence aorta staining showing more osteopontin (OPN)+ cells in male HSD and REV mice compared to their respective ND controls. **(D)** Aorta SMA-to- OPN ratio is lower in both male HSD and REV groups compared to ND controls.

*In panels A, B and D, values are given as median ± IQR; in panel C, values are given as mean ± SEM. * denotes P<0.05 after 2-Way ANOVA and Fisher's post-hoc testing.*

Figure S7. Lowering high dietary salt intake affects kidney gene expression.

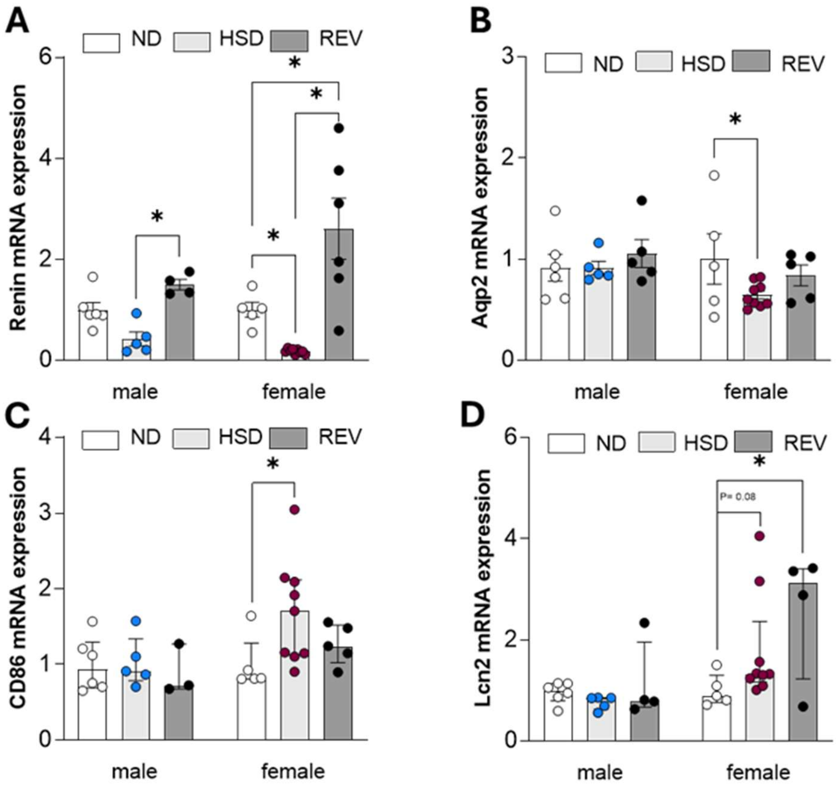

Kidney gene expression quantification showing **(A)** higher renin levels in the group with diet reversal (REV) compared to high salt diet (HSD) mice in both male and female sex, **(B)** higher aquaporin 2 (*Aqp2*) levels in the REV group compared to HSD in female mice, and **(C)** similar *CD68* levels in REV and ND groups in female mice. **(D)** *Lcn2* expression is higher in female HSD mice and further augmented after diet reversal.

*In panels A-B, values are given as mean ± SEM; in panels C-D, values are given as median ± IQR. * denotes P<0.05 after 2-Way ANOVA and Fisher's post-hoc testing.*

Figure S8. Lowering high dietary salt intake affects kidney structure.

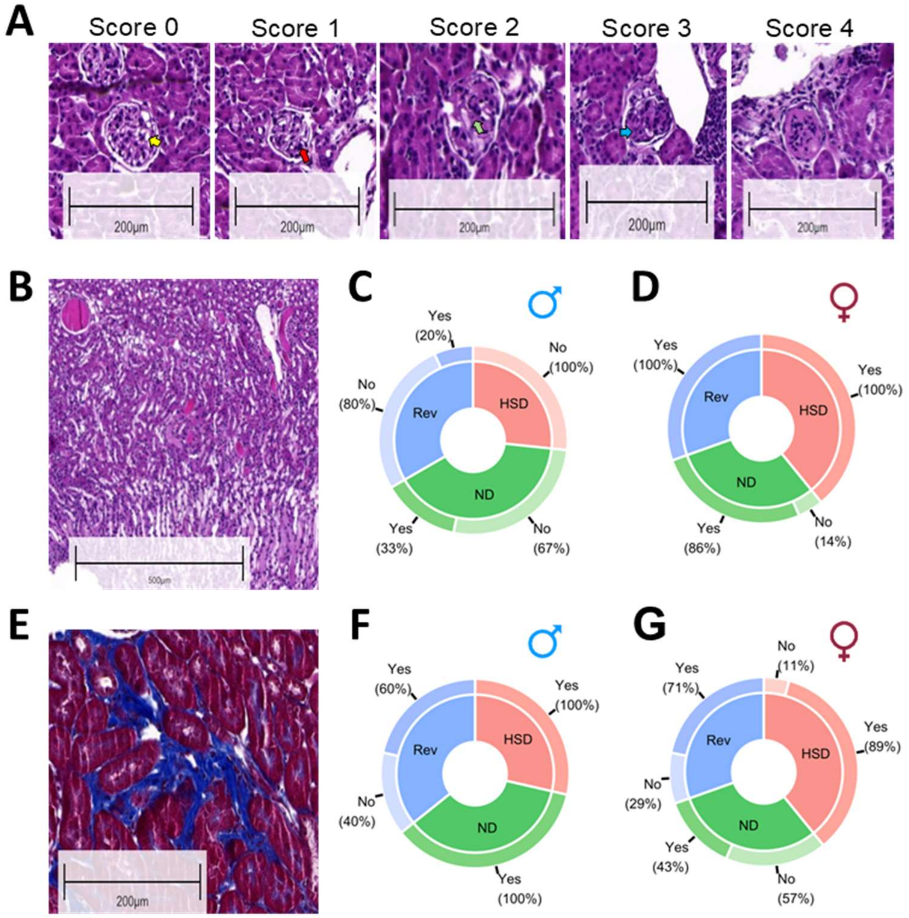

(A) Representative images depicting the extent of glomerular damage and the corresponding scoring criteria. Yellow arrow indicates a healthy, intact capillary; red arrow points to podocyte hypertrophy; green arrow denotes mesangial matrix expansion and blue arrow indicates glomerulosclerosis. Accordingly, score 0 represents a healthy glomerulus, score 1 corresponds to podocyte hypertrophy and mesangial matrix expansion, score 2 indicates podocyte hypertrophy, mesangial matrix expansion, and <50% sclerosis, score 3 represents >50% sclerosis, and score 4 indicates global glomerulosclerosis. **(B)** Representative image showing protein casts in the inner medulla. **(C)** Male mice show low to no age-related protein cast deposition in their renal tubules. **(D)** Female mice in the high salt diet (HSD), no diet (ND), and diet reversal (REV) groups demonstrate a higher incidence of protein casts compared to male mice. **(E)** Representative image showing the presence of tubular interstitial fibrosis. Male mice in the HSD and ND groups show a higher incidence of tubular interstitial fibrosis **(F)** than their female counterparts, while female mice in the Rev group exhibit a higher incidence of fibrosis than males **(G)**.

Figure S9. Lowering high dietary salt intake did not affect thickness of kidney vessel with diameters between 40-80 µm.

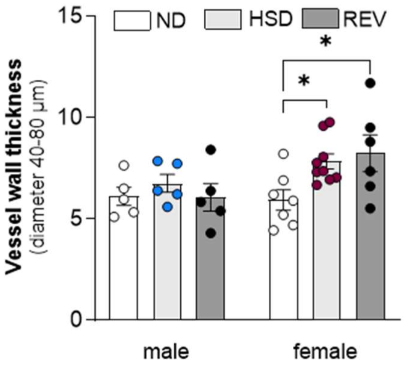

In female but not male high salt diet (HSD) mice, renal vessel thickness is enlarged in vessels with diameters between 40-80 µm. Diet reversal (REV) did not affect this response.

*Values given as mean ± SEM. * denotes P<0.05 after 2-Way ANOVA and Fisher's LSD post-hoc testing.*
